# α9-Containing Nicotinic Receptors in Cancer

**DOI:** 10.3389/fncel.2021.805123

**Published:** 2022-01-21

**Authors:** Susanna Pucci, Michele Zoli, Francesco Clementi, Cecilia Gotti

**Affiliations:** ^1^Institute of Neuroscience, National Research Council (CNR), Milan, Italy; ^2^NeuroMi Milan Center for Neuroscience, University of Milano Bicocca, Milan, Italy; ^3^Department of Biomedical, Metabolic and Neural Sciences, Center for Neuroscience and Neurotechnology (CfNN), University of Modena and Reggio Emilia, Modena, Italy; ^4^Department of Medical Biotechnology and Translational Medicine, Università degli Studi di Milano, Milan, Italy

**Keywords:** nicotinic acethylcholine receptor (nAChR), cancer, lung, breast, glioblastoma, melanoma

## Abstract

Neuronal nicotinic acetylcholine receptors containing the α9 or the α9 and α10 subunits are expressed in various extra-neuronal tissues. Moreover, most cancer cells and tissues highly express α9-containing receptors, and a number of studies have shown that they are powerful regulators of responses that stimulate cancer processes such as proliferation, inhibition of apoptosis, and metastasis. It has also emerged that their modulation is a promising target for drug development. The aim of this review is to summarize recent data showing the involvement of these receptors in controlling the downstream signaling cascades involved in the promotion of cancer.

## Introduction

There is ample evidence that the risk of developing various types of cancer is greater among smokers than non-smokers (Grando, 2014), although the role of smoking in the etiology of some cancers remains controversial (Li et al., 2016; Shao et al., 2016). Tobacco smoke contains at least 4,000 compounds (nicotine and a number of carcinogenic compounds such as tobacco-specific nitrosamines, polycyclic aromatic hydrocarbons, and aldehydes) that are capable of inducing the DNA damaging response that initiates tumorigenesis and enhances the spread of metastases. The risk and severity of cancer depend on the duration of exposure and the amount of tobacco smoking (Schane et al., 2010).

Nicotine is the most addictive component of tobacco smoke and, although it is unable to initiate tumorigenesis in humans and rodents, it promotes tumor growth and metastasis by inducing cell-cycle progression, migration, epithelial-to-mesenchymal transition, invasion, angiogenesis, and the evasion of apoptosis in a variety of systems (Schaal and Chellappan, 2014; Mucchietto et al., 2016), and ultimately causes multiple cancers to develop resistance to chemotherapeutic drugs (Grando, 2014; Afrashteh Nour et al., 2021). Emerging evidence suggests that there is a pathogenic link between nicotine and an increased susceptibility of cells to malignancies, but the underlying mechanisms are still ill-defined. The primary mechanism underlying the way in which nicotine acts as a tumor promoter is by binding and activating nicotinic acetylcholine receptors (AChRs; Mucchietto et al., 2016), which induce the secretion of the growth factors and cytokines that alter the physiology of various organ systems and can activate a number of intracellular mitogenic signaling pathways to promote cell growth, angiogenesis, and other tissue responses. These receptors represent a highly heterogeneous class of ligand-gated ion channels that are enriched in skeletal muscle (where they transduce nerve-to-muscle communication) and in the central and peripheral nervous systems, where they mediate synaptic transmission (Hurst et al., 2013; Zoli et al., 2015).

Neuronal nicotinic acetylcholine receptors (nAChRs) are a family of subtypes that are mainly heteromeric combinations of nine α (α2-α6) and three β (β2-β4) subunits, or homomeric receptors consisting of α subunits (α7 or α9); these last subunits can also form the heteromeric α7β2 and α9α10 subtypes (Zoli et al., 2015). nAChRs are activated by endogenous ligands such as ACh and choline, and exogenous compounds such as nicotine. The α-bungarotoxin (αBgtx) antagonist binds and blocks only some of the nAChR subtypes, which are therefore divided into two main classes: the αBgtx-sensitive subtypes, which may be homomeric or heteromeric and consist of the α7, α7β2, α9, and α9α10 subtypes, and the αBgtx-insensitive receptors, which are always heteromeric combinations of α (α2-α6) and β (β2-β4) subunits (Gotti et al., 2006; Albuquerque et al., 2009).

Recent studies have clearly shown that nAChRs and their physiological agonist, ACh, are widely expressed in non-neuronal mammalian cells (Grando, 2014; Zoli et al., 2018; Chen et al., 2019) from which ACh is released into the extra-cellular space in an autocrine, paracrine or juxtacrine manner, and that the cholinergic/nicotinic system plays important roles in various aspects of cell biology and homeostasis (Wessler and Kirkpatrick, 2008; Friedman et al., 2019). nAChRs and their endogenous ligands are also expressed by cancer cells and tissues.

### Structure, Pharmacology, and Localization of α9 nAChRs

The various combinations of nAChR subunits lead to the formation of a heterogeneous family of pentameric receptor subtypes that have different structural, functional, and pharmacological properties. In heteromeric nAChRs consisting of α and ß subunits, the ACh binding site (also known as the orthosteric site) lies at the interface of the extra-cellular domains of adjacent subunits and consists of a principal component of one α subunit, and a complementary component of the adjacent ß structural subunit.

The α9α10 subtype is a heteromeric receptor that only consists of α subunits (α9 and α10), and it has been proposed that α10 may act as a structural subunit, in much the same way as a β subunit of heteromeric nAChRs providing only complementary components to the agonist binding site (Elgoyhen and Katz, 2012). However, recent studies have shown that α9 and α10 subunits equally contribute to the principal component of the α9α10 nAChR binding site, whereas the contribution of α9 and α10 to the complementary component is non-equivalent (Boffi et al., 2017).

Experiments on the X-ray crystal structures of the extracellular domain of the monomeric state of the human neuronal α9 subunit and its complexes with a αBgtx, methyllycaconitine (MLA; Zouridakis et al., 2014) or the α-conotoxin RgIA (Zouridakis et al., 2019) have shown that, despite the absence of a complementary subunit and transmembrane domain, the α9 extracellular domain has a native folding and can bind MLA, αBgtx or α-conotoxin RgIA through its principal component.

The α9α10 subtype may have two different stoichiometries, (α9)_2_(α10)_3_ (Plazas et al., 2005) and (α9)_3_(α10)_2_ (Indurthi et al., 2014), which have varying sensitivities to ACh and the Vc1.1 α-conotoxin. In addition to the computationally established α9α10 binding site common to both stoichiometries, the (α9)_3_(α10)_2_ stoichiometry has an additional, binding site at the α9/α9 interface of low sensitivity for the α-conotoxin Vc1.1 (Indurthi et al., 2014).

On the basis of their sequence similarity to nAChRs, the α9 and α10 subunits were initially classified as neuronal, but the homomeric α9 and the heteromeric α9α10 receptors are not functionally present in the brain (Morley et al., 2018). The α9α10 subtype is only endogenously expressed in cochlear and vestibular hair cells in the inner ear, and in extra-neuronal tissues. Studies of α9 (Vetter et al., 1999) and α10 knockout (KO) mice (Vetter et al., 2007) have demonstrated the physiological role of this subtype in the medial olivocochlear pathway, in which the targeted disruption of the α9 (Vetter et al., 1999) or α10 subunits (Vetter et al., 2007) abolishes ACh-mediated synaptic currents in both inner and outer hair cells. More recently, Morley et al. (2017) have used constitutive α9 and/or α10 KO mice to show that only the doubly constitutive mice showed abnormal efferent innervation of cochlear hair cells. Moreover, a very recent study (Wang et al., 2021) has demonstrated that, in the developing auditory system, α9 and α10-containing receptors are involved in the spontaneous activity generated in the cochleae that propagates to the CNS to promote circuit formation.

α9 -containing receptors are also expressed at the splanchnic nerve–chromaffin cell synapse, where they are involved in the excitatory neurotransmission. These receptors are upregulated in cold-stressed rats where they become a major determinant of ACh-induced current (Colomer et al., 2010).

α9 subunit is also expressed by the cells of the immune system (Lustig et al., 2001; Hecker et al., 2015) but unlike in neurons and cochlear hair cells, where α9-containing receptors function as ligand-gated ion channels, in immune cells α9 ligand-evoked ion currents have not been observed (Richter et al., 2016).

The functional properties of α9α10 nAChRs vary widely across species (Marcovich et al., 2020). *Xenopus* oocytes expression of the mammalian α9 subunit forms functional channels, whereas the rat or human α10 subunit only forms functional channels when it is co-expressed with an α9 subunit. The oocytes co-injection of α9 and α10 subunits leads to a subtype that has the same ACh EC_50_ as the α9 subtype but greater functional expression (Elgoyhen et al., 1994, 2001).

A number of the pharmacological characteristics of α9 and α9α10 nAChRs are particularly interesting: they are activated by ACh but not by the classical agonist nicotine that blocks ACh-gated currents, and the α9α10 nAChR has a mixed nicotinic and muscarinic profile as it is blocked by the nicotinic antagonists d-tubocurarine and αBgtx as well as by the muscarinic antagonist atropine (Elgoyhen et al., 1994, 2001; Sgard et al., 2002).

### nAChR-Associated Proteins

All nAChRs are integral membrane proteins that share the same basic topology, and neurotransmitter receptors coupled to an ion-conducting pore. A plethora of proteins and metabolites acting at multiple steps in nAChR biogenesis have recently been discovered (Crespi et al., 2018; Matta et al., 2021): some of these proteins are endoplasmic reticulum-resident chaperone proteins that are necessary for the proper folding and assembly of some nAChR subtypes, whereas others are auxiliary (accessory) subunits that stably associate with receptors and control channel function.

When expressed in transfected cell lines, the α9α10 subtype is not functional. Genome-wide screening has shown that choline acetyltransferase (ChAT), the biosynthetic enzyme for ACh, and the protein TMIE (Gu et al., 2020) are factors enabling the function of α9α10 nAChRs. Functional studies have demonstrated that the assembly and surface expression of α9α10 nAChRs require the ligand binding of an agonist (ACh) or antagonist (αBgtx), and that mutating the α9α10 ACh binding site abolishes receptor surface expression. They have also shown that the presence of TMIE protein, a deafness gene product, is important for the correct functioning of α9α10 nAChRs: TMIE acts as an auxiliary subunit and, by associating with the α9α10 receptors on the cell surface, promotes their function (Gu et al., 2020).

The proteins that stably associate with nAChRs comprise the LY6 prototoxin family, a family of small modulatory proteins that can interact with numerous targets. These proteins have a three- dimensional structure that is similar to that found in snake venom neurotoxins (Miwa, 2021). Some of them are secreted as water-soluble proteins, and others are bound to cell membranes by a glycosylphosphatidylinositol (GPI) anchor. Lynx1, the first member of the LY6 family to be identified, can form a stable complex and regulate the responses of α4β2 and α7 nAChRs. Lynx1 is expressed in normal and neoplastic lung tissue, but its level is lower in lung cancers than in adjacent normal lung tissue (Fu et al., 2015). The recombinant water-soluble variant of Lynx1 inhibits the growth of adenocarcinoma A549 cells by causing cell cycle arrest *via* the modulation of α7 nAChRs and the activation of various intra-cellular signaling cascades (Bychkov et al., 2019). Although α9α10 nAChRs have not been detected among the targets of Lynx1, it has been shown that a recently synthesized circularized Lynx1-derived peptide binds to the ligand binding domain of α9 nAChRs and inhibits the rat α9α10 subtype expressed in *Xenopus* oocytes (Kryukova et al., 2019).

### Ionotropic and Metabotropic nAChR Signaling

The ionotropic mechanism of inter-cellular signaling mediated by nAChRs has been studied in neurons (Albuquerque et al., 2009; Corringer et al., 2012), and is due to the opening the ionic channel that is an integral part of the receptor and generating cation fluxes that lead to membrane depolarization. This depolarization opens voltage-gated Ca^2+^ channels (VGCCs), and leads to a further Ca^2+^ influx that may induce Ca^2+^-induced Ca^2+^ release (CICR) from the endoplasmic reticulum by activating ryanodine receptors (RyRs; King et al., 2015; Kabbani and Nichols, 2018). The nAChR subtypes with the greatest Ca^2+^ permeability are the homomeric α7 and α9, and the heteromeric α9α10 nAChRs (Fucile et al., 2003, 2006).

In non-excitable cells, the increase in the concentration of free intracellular Ca^2+^ induced by activation of nAChRs mainly derives from ionic influx through the receptors since VGCCs are poorly expressed.

In astrocytes, whose membrane resting potential is critically lower than that measured in neurons, ionic influx through ligand-gated channels may be largely responsible for the increased intracellular Ca^2+^ levels that modulates Ca^2+^-gated channels locally, and activates various Ca^2+^-dependent signaling mechanisms (Mcneill et al., 2021).

However, over the last decade, it has been shown that α7-, α4-, β2-, and β4-containing nAChRs also have a metabotropic mechanism underlying the neuronal response to nicotinic agonists (Nordman and Kabbani, 2014; King et al., 2015). The metabotropic pathway increases intracellular [Ca^2+^] *via* Gαq protein coupled with nAChR signal transduction mediated by phospholipase C (PLC) and inositol-trisphosphate (IP_3_) receptor-activated Ca^2+^ release from the endoplasmic reticulum (King et al., 2015; Kabbani and Nichols, 2018).

There are new lines of evidence that, in neurons, agonist stimulation of presynaptic α7 nAChRs mediates Ca^2+^ signaling *via* both ionotropic Ca^2+^ influx through the open-channel state and non-conventional metabotropic channel signaling. Agonist binding to α7 determines sufficient Na^+^ influx-induced depolarization to activate VGCCs and Ca^2+^ influx and lead to RyR-mediated CICR from the endoplasmic reticulum. In addition, the transition of α7 nAChRs to a high-affinity ligand-bound desensitized state supports a metabotropic response marked by G protein activation of PLC, IP_3_ production, and IP_3_-induced Ca^2+^ release (Papke and Lindstrom, 2020; Borroni and Barrantes, 2021).

Kabbani’s group (King et al., 2015; King and Kabbani, 2016; Kabbani and Nichols, 2018) has shown that the α7 subunit binds Gα and Gßγ proteins through the intracellular M3-M4 loop, and enables a downstream Ca^2+^ signaling response that can persist longer than the expected time course of channel activation and leads to the activation of downstream second messenger pathways such as RhoA GTPase and cytoskeletal changes during neurite growth.

In many non-neuronal cell types, metabotropic signaling after ligand binding is a property of α7 receptors and it is present in immune cells where cholinergic signaling plays an important role in regulating cytokine release even though no ACh-dependent currents have been recorded, and despite the fact that α7 receptors are on the plasma membrane (Fujii et al., 2017; Treinin et al., 2017).

It has also been shown that α9 subunit-containing nAChRs have metabotropic functions. Homomeric α9 nAChRs or heteromeric α9α10 nAChRs heterologously expressed in *Xenopus* oocytes do not generate ion currents when stimulated with phosphocholine, but do so when stimulated by choline, a metabolite of ACh (Richter et al., 2016) that also acts as an agonist of the α7 nAChR.

Various metabotropic functions of α7 and α9 nAChRs have been described in monocytes, in which choline derivatives (choline, phosphocholine, and ACh) inhibit ATP-mediated IL1ß release by blocking purinergic P2X7 receptors (Hecker et al., 2015). The effect of phosphocholine on IL-1ß release takes place *via* activation of the α9α10 metabotropic pathway. ACh and phosphocholine both inhibit IL-1ß release in monocytes, but only the former evokes ion currents through the receptor. Pre-incubation with phosphocholine reduces ACh-evoked ion flux, thus suggesting that phosphocholine acts as a silent agonist that converts α7 or α9 receptors to a long-lived desensitized state that elicits signal transduction independently of ion channel opening (Richter et al., 2016; Papke and Lindstrom, 2020).

In brief, depending on the type of cell and the nAChR subtypes expressed, the binding of ACh or nicotine can induce conformational changes in nAChRs and/or their associated proteins that can activate various intracellular signaling pathways and regulate gene expression.

### Methods Used to Study α9-Containing Receptors

A number of approaches have been used to detect nAChRs in many extra-neuronal tissues. Reverse transcription polymerase chain reaction (RT-PCR), quantitative RT-PCR, and *in situ* hybridization have all been used to show that they are expressed at variable levels in normal and malignant cells, but interpreting the data obtained is not easy because there is not always a correlation between the levels of mRNA and those of the expressed subunit.

The first evidence of α9 nAChR expression came from rat cochlear hair cells, in which α9 nAChRs are involved in auditory functions (Elgoyhen et al., 1994), but many more locations and biological functions of α9 nAChR have since been identified, including keratinocyte adhesion (Nguyen et al., 2000; Chernyavsky et al., 2007), breast epithelial cancer formation (Lee et al., 2010), endocrine activities (Colomer et al., 2010), immune responses (Hecker et al., 2015), inflammation (Liu et al., 2017, 2019), chronic pain (Romero et al., 2017; Hone et al., 2018; Zheng et al., 2021), the homeostasis of osteocytes, and the regulation of bone mass (Baumann et al., 2019).

Supplementary Table 1 summarizes the studies in which the presence of α9- and α9α10-containing receptors has been shown in cancer cells and tissues.

The most convincing identification of α9 nAChRs in such tissues has been obtained by the use of subtype-specific antagonists (Li et al., 2021) that block many cancer-promoting processes and/or by comparing their effects on the wildtype (WT) nAChR subunit and KO mice. It is important to note that the antagonists αBgtx and MLA not only act on α9 nAChRs but also bind with high affinity to α7 nAChRs (Elgoyhen et al., 1994; Verbitsky et al., 2000). Consequently, if they are used to study the *in vivo* or *in vitro* effects of nicotine or ACh on native α9 nAChRs, it needs to be remembered that α7 nAChRs may also be involved. In order to differentiate or analyze the effects mediated only by α9 nAChRs, the further use of α9-selective antagonists such as α-conotoxin Vc1.1 (Indurthi et al., 2014), α-O-conotoxin GeXIVA (Luo et al., 2015), RgIA4 (Romero et al., 2017), and PeIA (Mcintosh et al., 2005; Yu et al., 2018) is crucial. Dimeric constructs of the toxins PeIA, Vc1.1, and RgIA# ([ΔR13]RgIA) have recently been designed, and all three constructs are more potent than their monomeric counterparts in relation to human α9α10 nAChRs (Liang et al., 2020; see Table 1).

**Table 1:** Alkaloid and peptidic antagonists targeting α9-containing receptors.

Antagonists	Target subtypes	References
Methyllycaconitine	α6, α7, α9, α9-α10	Davies et al. (1999), Verbitsky et al. (2000), Elgoyhen et al. (2001), Zoli et al. (2002), and Fucile et al. (2006)
αBungarotoxin	α7, α9, α9-α10	Couturier et al. (1990), Elgoyhen et al. (1994), and Elgoyhen et al. (2001)
α-conotoxin PeIA	α9-α10	Mcintosh et al. (2005) and Yu et al. (2018)
αconotoxin RgIA	α9-α10	Ellison et al. (2006)
α-conotoxin Vc1.1	α9-α10	Indurthi et al. (2014)
α-O-conotoxin GeXIVA	α9-α10	Luo et al. (2015)
α-conotoxin RgIA4	α9-α10	Romero et al. (2017)
Dimeric PeIA, Vc1.1 and RgIA# ([ΔR13]RgIA)	α9-α10	Liang et al. (2020)

Another successful approach to studying the *in vitro* and *in vivo* roles of α9- and α10-containing receptors is to over-express or silence them by RNA interference, a very powerful technique that has given important information concerning the involvement of these receptors in physiological and pathological conditions (Lee et al., 2010).

Extensive work has very recently been done to identify human cancer proteins interacting with membrane proteins and determine their downstream regulated pathways using a systematically integrated method. As α9 is an integral membrane protein, Lin et al. (2019) have tried to identify new α9 nAChR interacting and downstream proteins by means of multiple RNA-seq and microarray data relating to different cancers. Using *CHRNA9*, they identified 18 new representative candidates that were subsequently verified by means of immunoprecipitation and Western blotting. They also found associated proteins that are important for regulated pathways, such as proteins involved in the cell cycle, adherens junctions, and ErbB signaling. These were related to cell growth and communication in more than six cancer types, thus indicating that α9 plays a role in the formation, progression, and metastatic spread of various tumors (Lin et al., 2019).

As recent exhaustive reviews (Schuller, 2009; Grando, 2014; Schaal and Chellappan, 2014) have already considered the mechanisms by means of which nAChRs are involved in cancer signaling pathways, this review will concentrate on the latest published data concerning the role of α9/α9α10 (α9*) receptors in the physiology and pathology of lung, breast, glioblastoma, and melanoma cancers.

### α9-Containing Receptors in Lung Cancer

Lung cancer is the most frequent and lethal cancer in the world, accounting for 1.79 million deaths in 2020 (Ferlay et al., 2021) and, although a subset of patients develop lung cancer without a history of smoking, approximately 70% of all cases of non-small cell lung cancer (NSCLC) and 90% of all cases of small cell lung cancer (SCLC) are caused by smoking (Samet et al., 2009; Pesch et al., 2012; Improgo et al., 2013).

A very recent study (Zhang et al., 2020) has shown that nicotine treatment of human lung epithelial cells can create a pro-tumorigenic environment favoring cell transformation or tumorigenesis in lung epithelium by perturbing intracellular redox status and altering p53 function. Together with a study by Tang et al. (2019) showing that mice exposed to nicotine delivered by means of electronic cigarette smoke develop lung adenocarcinoma, this suggests the need for caution when using nicotine replacement therapies and e-cigarettes.

Lung cells express the mRNA of multiple nAChR subunits whose levels are frequently altered in primary NSCLC and SCLC, and their cell lines. It has been found that the levels of α3, α5, ß4, and α9 subunit mRNAs are significantly increased in SCLC (Improgo et al., 2010, 2013), and that the levels of α7, α9, and α10 subunit mRNA are also increased in a number of cell lines derived from SCLC and NSCLC tumors (Improgo et al., 2010).

Various studies have shown that the nAChRs present in lung epithelial and endothelial cells play a direct role in lung cancer. In particular, nAChRs are expressed in airway epithelium cells that synthesize, store, and secrete the physiological ligand ACh, which acts as an autocrine and paracrine growth factor (Spindel, 2016; Friedman et al., 2019). When lung cancer arises from the airway epithelium, cell growth is stimulated by ACh or nicotine, and this may provide endogenous mitogenic signaling without any further activation.

Inhibiting cholinergic signaling causes apoptosis in bronchioalveolar carcinoma cells (Lau et al., 2013), and nicotine protects lung cancer cells against apoptosis induced by chemotherapy (Liang et al., 2020), oxidative stress, and ionising radiation (Friedman et al., 2019).

Case control studies of NSCLC patients (340) and matching controls (435) have shown that an increased risk of developing lung cancer is associated with two single nucleotide polymorphisms (SNPs) in the *CHRNA9* gene: rs56159866 and rs6819385 (Chikova et al., 2012).

NSCLC can be subdivided into adenocarcinomas, squamous cell carcinomas, and large-cell lung carcinomas. Adenocarcinomas are the most frequent type of NSCLC and develop from small airway epithelial cells and alveolar type II cells, whereas squamous cell carcinomas arise from large airway epithelial cells (Meza et al., 2015), and α7 and α9 mRNAs have been detected in adenocarcinoma and squamous carcinoma cells, as well as in human tumor samples in which their levels were higher than those observed in normal lung tissue (see Supplementary Table 1).

### α9-Mediated Signaling Pathways in Lung Tumors

Studies of A549 adenocarcinoma cells have shown that treatment with a nicotine concentration that is similar to the levels measured in sera of smokers (100 nM) promotes their proliferation (Mucchietto et al., 2018), and this proliferation is blocked by the action of MLA and αBgtx on α7 and α9 receptors, and by subtype-specific α9 (RgIA4) and α7 toxins (AR). The concentration of nicotine increasing cell proliferation was much less than that required to activate α9 or α7 receptors in functional experiments. This suggests that nicotine induced the non-ionic signaling events that regulate the phosphorylation states of the target proteins mediating some of the effects of nicotine on A549 cells (Schaal and Chellappan, 2014). In the same cells, nicotine acting on α9-containing receptors stimulates the Akt and ERK cell signal transduction pathways, and the use of the α9-selective peptidic antagonist RgIA4 prevents nicotine-induced ERK and Akt phosphorylation The involvement of α9-containing nAChRs in nicotine-induced proliferation and intracellular signaling in A549 cells has been further confirmed by the fact that silencing experiments using siRNA directed against α9 mRNA replicated the results obtained using RgIA4. All the effects of nicotine on A549 cell proliferation and signaling activation described above are also blocked by the α7-selective toxin AR and the α7- and α9-unselective antagonists MLA and αBgtx, thus suggesting that both receptors have to be activated in order to reach the cytoplasmic Ca^2+^ concentration that triggers the biological effects. In addition, the fact that the nicotine-induced proliferation and intracellular signaling of A549 cells is blocked by α7 and α9 peptidic antagonists that are not cell permeable indicates that the effects of nicotine are due to the activation of the nAChRs present on the plasma membrane.

### α9-Containing Receptors in Breast Cancer

In 2020, it was estimated that breast cancer accounted for about 2.26 million new cases and 682, 000 deaths, thus making it the most frequently diagnosed cancer worldwide, (Ferlay et al., 2021). It is also the second leading cause of cancer-related deaths among women, mostly of which are due to recurrences and metastases (Ferlay et al., 2021).

Tobacco smoke may contribute to a woman’s risk of developing breast cancer as large-scale cohort studies conducted in the United States and Japan indicate that the risk is associated with both active and passive smoking (Slattery et al., 2008).

One case-control study of 737 breast cancer patients and 719 age-matched healthy controls has studied the association between *CHRNA9* SNPs and the risk of breast cancer, and examined the joint effect of cigarette smoke exposure and *CHRNA9* SNPs on developing breast cancer. It found that an increased risk of breast cancer is associated with the variant rs73229797 allele on the *CHRNA9* gene, and that the risk is greater in both active and passive smokers (Hsieh et al., 2014).

Over the last decade, it has been shown that α9 nAChRs are important in the formation of breast cancer. Lee et al. (2010) have shown that α9-containing nAChRs are expressed in many, breast cancer, epithelial and lung cells most of which also express α10 and α5 subunits. They also found that α9-containing nAChRs are present in both primary tumors and non-malignant breast tissue obtained from patients (see Supplementary Table 1), but their expression is higher in breast cancer cells than in the surrounding normal tissue. Not only did 67.3% of their 276 breast cancer tissue samples express high α9 nAChR levels, this expression was higher in the advanced stage III and IV samples than in the early stage I and II samples, and this occurred more often in smokers than in passive smokers or non-smokers.

### α9-Mediated Effects of Nicotine on Breast Cancer Cells

In order to explore the potential carcinogenic effects of the α9 subunit expressed in normal human breast epithelial cells, Lee et al. (2010) established the MCF-10A normal human breast cell line with the tetracycline-regulated (Tet-off) over-expression of α9 nAChRs, and found that it became potentially carcinogenic *in vitro* and *in vivo*. Chen et al. (2011) found that the same line xenografted in BALB/c-nu/nu mice induced cyclin D3 over-expression after exposure to nicotine, whereas silencing α9 nAChR expression in breast cancer cells reduced *in vitro* and *in vivo* cyclin D3 levels, cell proliferation, and tumorigenic potential (Chen et al., 2011).

It has been shown that 10 μM nicotine stimulates nAChRs (particularly α9-containing nAChRs) to induce proliferation in two breast cancer cell lines (MDA-MB 231 and MCF-7), and induces the growth of tumors xenografted into SCID mice (Lee et al., 2010). In addition, prolonged exposure of normal MCF-10A human breast to 10 μM nicotine or 1 μM 4-(methylnitrosamino)-1-(3-pyridyl)-1-butanone (NNK) triggers pre-cancerous transformation, an effect that was potentiated by increasing the expression of α9 nAChRs (Lee et al., 2010, 2011). It has also been shown that in MCF-7 human breast cancer cells, nicotine and estrogen induce α9 nAChR expression and that this is transcriptionally regulated by estrogen receptors (ERs). The binding of nicotine to α9-containing nAChRs activates the downstream PI3K, Akt or MAPK signaling pathways, thus facilitating the phosphorylation of ERs and their binding to the AP1 transcription factor and the *CHRNA9* promoter region.

**Figure 1:**
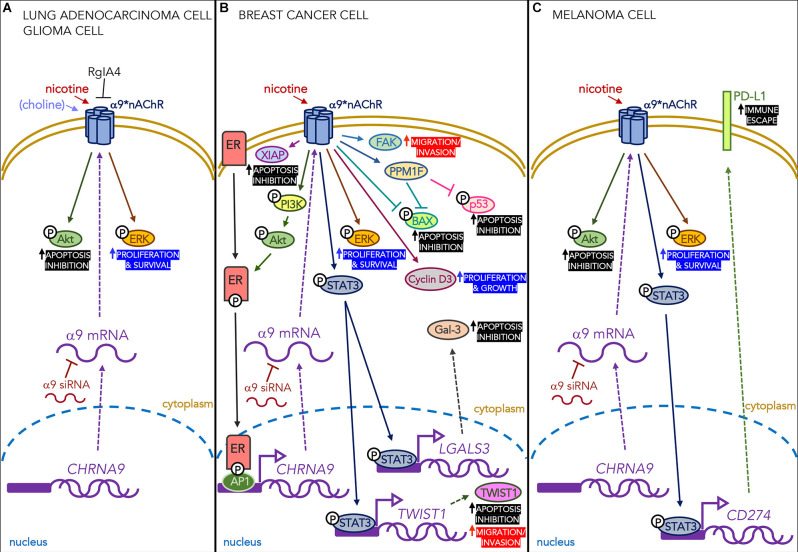
Effects of nicotine acting on α9-containing receptors expressed in different types of cancer cells. **(A)** Nicotine (in lung adenocarcinoma and glioma cells) and choline (only in glioma cells) mediate proliferative and anti-apoptotic effects through the ERK and Akt signaling pathways. These effects are blocked by the α9-selective antagonist RgIA4 and α9 siRNA. **(B)** In breast cancer cells, nicotine acting on α9 nAChRs activates the PI3K/Akt pathway that phosphorylates intra-cellular estrogen receptors (ERs) which, in their turn, bind transcription factor AP1 and the *CHRNA9* gene promoter region. It also increases the levels of pro-proliferative cyclin D3, the anti-apoptotic protein XIAP, and the PPM1F protein phosphatase that reduces phosphorylated p53 and BAX, thus protecting the cells from apoptosis. Finally, it promotes the phosphorylation of STAT3 and its nuclear translocation, thus increasing the transcription of the gene encoding the anti-apoptotic lectin Gal-3. All of these effects are blocked by α9 siRNA. **(C)** In melanoma cells, nicotine increases the number of α9 nAChRs and phosphorylates STAT3, which binds the *PD-L1* promoter in the nucleus and leads to the higher surface expression of PD-L1, thus promoting the immune escape of cancer cells. The increased expression of PD-L1 protein is down-regulated by α9 siRNA.

Nicotine exposure promotes the apoptosis resistance of breast cancer cells by increasing α9 expression, which activates STAT3 nuclear translocation and physical interactions with the promoters of the gene coding for Galectin-3 (*LGALS3*), an intra-cellular anti-apoptotic α-galactoside-binding lectin (Guha et al., 2014; see Figure 1), and the *TWIST1* promoter. Activated STAT3 directly binds these promoters, thus inducing their transcription and up-regulation, and this delays apoptosis and enriches a sub-population of breast cancer cells that have stem cell-like properties.

As it is still debated whether exposure to second-hand smoke (SHS) affects the risk of breast cancer, Fararjeh et al. (2019) used a model of breast cell carcinogenesis in which immortalized normal human breast epithelial cells exposed to a carcinogen undergo long-term exposure to 1 pM nicotine and/or 100 femtM NNK doses (that can be reached during exposure to SHS). They found that repeated co-exposure to nicotine and NNK disrupted cell growth control and led to the acquisition of cancer cell properties, and increased the levels of a number of α9 nAChR downstream signaling proteins, such as the FAK migration marker and the PI3K/Akt intracellular signaling pathway. They also found the increased expression of XIAP (a protein that may play an important role in breast cancer pathogenesis by inhibiting apoptosis) and a decrease in the expression of the pro-apoptotic protein BAX.

Cell invasion and the spread of metastases are crucial processes in tumor development that are regulated by numerous factors, including growth factors, phosphatases, kinases, and extracellular matrix components. PPM1F is a Ser/Thr protein phosphatase that is overexpressed during breast tumor invasion: the levels of PPM1F protein are higher in invasive MDA-MB-231 breast cancer cells than in non-invasive MCF-7 cells, and PPM1F levels are higher in the breast tumor tissue of smokers than in that of non-smokers. Nicotine induces *in vitro* PPM1F expression, whereas silencing α9 nAChRs reduces PPM1F protein levels, thus ultimately reducing cell motility and invasiveness. Tu et al. (2016) have found that the overexpression of PPM1F reduces the level of phosphorylated p53 and phosphorylated BAX in nicotine-treated MDA-MB-231 cells. These findings show that PPM1F is a phosphatase that inactivates pro-apoptotic proteins (such as BAX) and proteins involved in cell cycle arrest (such as p53), and may be a downstream mediator of α9 nAChRs.

It has recently been shown that breast cancer cell proliferation *in vitro* can be inhibited by selectively antagonising α9α10 nAChRs (Sun et al., 2020) using the selective αO-conotoxin GeXIVA.

### α9 nAChRs in Triple-Negative Breast Cancer

Breast cancer negative for ERs, progesterone receptors, and excess human epidermal growth factor receptor 2 protein (triple-negative breast cancer, TNBC) is responsible for 15–20% of all breast cancer cases (Lee et al., 2019).

The over-expression of α9 nAChRs is associated with the poor 5-year disease-specific survival of patients diagnosed as having breast cancer as it up-regulates ER expression.

Huang et al. (2017) used a serial spontaneous pulmonary metastasis animal model to generate highly metastatic TNBC cells derived from metastasized lung cells, and found that there was a significant induction of *CHRNA9* gene expression and that the level of α9 nAChR expression correlated with the metastatic ability of the tumor. These findings suggest that α9 nAChR expression is required to mediate TNBC metastasis during the development of cancer, and may be a biomarker for targeted therapy.

### α9-Containing Receptors in Gliomas and Glioblastomas

Gliomas represent 81% of all primary malignant brain tumors in adults, and are one of the most aggressive human cancers (Jemal et al., 2011). The World Health Organisation (WHO) stratifies gliomas into grades I-IV, the most aggressive of which are grade IV astrocytomas or glioblastomas (GBMs; Louis et al., 2001). The global incidence of GBMs is 2–5 per 100,000 people and has increased over the last decade (Dobes et al., 2011). Median patient survival is approximately 14–17 months (Zhu et al., 2017), and the 5-year survival rate is only 5.1% (Ostrom et al., 2015). GBMs originate in brain neural cells and are highly proliferative and invasive. They may begin in astrocytes, glial precursor cells or neural stem cells, but the pathophysiological and molecular mechanisms underlying their malignant transformation are still unclear. The poor prognosis of GBM is primarily attributable to therapeutic resistance and tumor recurrence after surgical removal, which is due to the presence of a small sub-population of self-renewing and proliferative glioblastoma stem cells (GSCs) similar to the neural stem cells from which they may arise.

The results of numerous retrospective studies have failed to show any causal relationship between smoking and gliomas, which is why the hypothesis of a causative link between smoking and the etiology of malignant gliomas remains controversial (Hou et al., 2016; Li et al., 2016).

Very few articles have described studies of the mechanisms of action of nicotine on glioma or GBM cells and, given the limited amount of material available, this review will thoroughly discuss all of the most recent findings.

Khalil et al. (2013) have shown that nicotine concentrations comparable to those found in the serum of chronic smokers enhance GBM cell migration, proliferation, survival, clonogenicity, and radio-resistance partially by activating EGFRs, ß-adrenergic receptors, and the Akt and ERK signaling pathways, and that these effects can be blocked by pharmacological inhibitors of the same pathways. Nicotine also promotes tumor growth in nude mice xenografted with human malignant glioma cells, thus suggesting that nicotine exposure may enhance the growth of malignant glioma tumors *in vivo*.

Spina et al. (2016) correlated glioma patient survival with *CHRNA* gene expression using the Rembrandt portal^1^, and found that the survival of patients with gliomas or GBMs over-expressing *CHRNA1* or *CHRNA9* is significantly shorter. They used glial fibrillary acidic protein (GFAP) as a reporter of astrocyte differentiation and isolated multiple clones from three independent GSC lines that over-express *CHRNA1* or *CHRNA9* and express GFAP. Their results showed that high GFAP expression is associated with reduced clonogenicity *in vitro* and tumorigenicity *in vivo*. Their screening of chemical libraries revealed that the neuromuscular AChR blocker atracurium besylate inhibits the clonogenic capacity of patient-derived GSCs and induces astroglial differentiation, whereas a nicotinic receptor agonist prevents atracurium besylate from reducing the self-renewal of GSCs. This study also showed a significant improvement in the survival of mice xenotransplanted with GSCs that were pre-treated with atracurium besylate. Taken together, these findings suggest that α1- and α9-containing nAChRs may be intimately involved in controlling GSC fate by reducing their stemness and/or their population.

Thompson and Sontheimer (2019) have very recently investigated the expression of nAChRs and muscarinic receptors (mAChRs) in GBM datasets and cell lines, and patient-derived xenograft lines. Using the RNA-Seq data relating to the tumor samples taken from 156 GBM patients, they analyzed AChR mRNA expression and found that the expression of the large majority of subunits was low, whereas that of the muscle-type α1 and ß1 subunits, and the neuronal α9 subunit was high. They used the calcium time-lapse technique to show that the activation of mAChRs or nAChRs increases intra-cellular Ca^2+^ in GBM cells. They also showed that there is a nicotine-mediated increase in intracellular Ca^2+^ that is due to α7 receptors (and probably α9-containing receptors, which are highly permeable to Ca^2+^ and show increased mRNA expression in patient-derived xenograft GBM lines). In neurons, the time-course of nicotine and ACh activation of the homomeric α7 receptor is very fast (in the order of milliseconds; Papke and Lindstrom, 2020; Borroni and Barrantes, 2021), whereas in GBM cells nicotine activates Ca^2+^ influx with a time-course of hundreds of seconds. This Ca^2+^ signaling response persists longer than channel activation and may be the result of CICR from intra-cellular stores, as has been previously shown in the case of hippocampal astrocytes (Sharma and Vijayaraghavan, 2001). It was also demonstrated that the increase in intracellular Ca^2+^ induced by ACh has minimal effects on cell proliferation or migration, whereas the stimulation of both mAChRs and nAChRs increases GBM cell invasion due to the increased activity of metalloproteinase-9.

Our group has also been interested in characterizing the nAChRs in glioblastoma cells. We began by examining publicly available data (accession number GSE16805) from a large-scale, multi-dimensional DNA microarray analysis carried out at IRCSS AOU San Martino-IST (Genoa, Italy), which showed that the most frequently expressed nAChR subunit mRNAs were CHRNA5 and CHRNA9 and, to a lesser extent, CHRNA7 and CHRNA10. We then analyzed the *in vitro* presence and possible role of nAChRs in U87MG human WHO grade IV astrocytoma U87MG cells and the GBM5 cell line derived from CSC-enriched cultures resistant to temozolomide (TMZ) treatment (Banelli et al., 2015), and found that the expression of nicotinic subunits was similar to that recorded in public databases of GBM patients: highly expressed α9 and α5 subunit mRNAs and less expressed α7 and α10 subunit mRNAs.

The results of our study extended the findings of Khalil et al. (2013) as we found that chronic treatment with nM concentrations of choline or nicotine increases the proliferation of U87MG and GBM5 cells, and activates the anti-apoptotic Akt and pro-proliferative ERK pathways. In general, these effects were blocked by silencing the expression of the α7 and α9 subunits by means of siRNAs or by the non-cell-permeable peptide antagonists selective for α7- and α9-containing nAChRs, thus indicating that they required the presence of both subunits. However, the effects of α7 and α9 were not identical as the action of choline on U87MG and GBM5 cell signaling was not significantly reduced after silencing the α7 subunit, whereas nicotine-induced signaling and choline- and nicotine-stimulated cell proliferation were significantly prevented in both cell lines after silencing either of the subunits. This last finding led us to deduce that choline may act mainly through α9-containing nAChRs, at least in activating GBM5 cell signaling. The α9-containing nAChRs are also involved in basal GBM cell proliferation as it decreased after 6 days of treatment with a subtype-specific antagonist (Pucci et al., 2021).

The agonist concentrations that increased cell proliferation were much lower than those necessary to activate α7 (nicotine and choline) or α9 nAChRs (choline) in electrophysiological experiments (Elgoyhen et al., 1994; Corradi and Bouzat, 2016). As nicotine is an antagonist of the ionotropic effects of α9 receptors, the occupation of nAChRs in U87MG and GBM5 cells by low concentration of nicotine leads to a conformational change that may alter interactions between nAChR scaffolds and other intracellular signaling molecules or cytoskeletal elements. This may elicit the non-ionic, metabotropic signaling events that regulate the phosphorylation status of target proteins and thus mediates some of the effects of choline and nicotine.

In our experiments, blocking α7 and/or α9 nAChRs by means of the use of selective antagonists or siRNAs substantially abolished choline- and nicotine-induced cell proliferation and decreased the effects of phosphorylation, which again indicates that the presence of receptors containing both subunits is necessary to ensure the effects of nicotinic agonists. This may be because: (1) it is necessary to have both α7- and α9-containing nAChRs in order to ensure sufficient target cell activation; or (2) GBM cells express heteropentameric α7α9-containing nAChRs which are mainly responsible for the effects of nicotine and choline. Given the difference in the levels of α7 and α9 mRNAs (Pucci et al., 2021), the hypothesis of an α7α9-nAChR subtype may seem to be unlikely at first glance, but it cannot be excluded because the turnover of the two mRNAs may be different, and mRNA levels are not always representative of the expressed protein.

As choline is a precursor of ACh, the increase in GBM cell proliferation may have been due to the direct action of either compounds, but the blocking effect of α7/α9 nicotinic antagonists clearly indicates mediation by nAChRs. Thompson and Sontheimer (2019) found that the expression of the mRNAs of the proteins necessary for ACh synthesis and release (many choline transporters, choline acetytransferase, and vescicular acetylcholine transporters) is significantly higher in GBM tissues than in their non-tumoral counterparts, thus suggesting that ACh is the signaling molecule acting on nAChRs.

The α7 and α9α10 nAChRs in GBM cells are pharmacologically similar to those expressed in A549 lung adenocarcinoma cells, whose nicotine-induced activation also increases their proliferation (Mucchietto et al., 2018). This proliferation is blocked in a concentration-dependent manner, by the selective MG624 nicotinic ligand (Gotti et al., 1998). Hybridization of MG624 and a non-nicotinic, resveratrol-derived pro-oxidant mitocan (Bavo et al., 2018) has led to the development of two novel compounds (StN-4 and StN-8) that are more potent than MG624 in reducing the viability of GBM cells, but less potent in reducing the viability of mouse astrocytes (Bavo et al., 2018). Knocking down the α9 subunit in U87MG cells decreases the potency of the effects of both compounds on cell viability, thus clearly indicating that α9-containing nAChRs are involved in sustaining GBM cell growth and survival (Pucci et al., 2022).

### α9-Containing Receptors in Melanoma

Melanoma arises from genetic mutations in melanocytes, and is the most aggressive and deadly skin cancer mainly affecting Caucasians. It accounts for less than 5% of all skin cancers diagnosed annually in the United States, but is responsible for more than 75% of skin cancer deaths (Gershenwald and Guy, 2015).

Studies of 2,583 malignant melanoma patients indicate that smoking facilitates the spread of metastases in both men and women (Shaw and Milton, 1981), and a more recent study of a large sample of current smokers has found a close association between smoking and the occurrence of sentinel lymph node metastases (Jones et al., 2017).

Programmed death-ligand 1 (PD-L1) is a type 1 transmembrane protein encoded by the human *CD274* gene that is overexpressed in some types of cancer (Salmaninejad et al., 2019), and PD-1 is an inhibitory receptor encoded by the *PDCD1* gene that is located on the surface of all T cells (LaFleur et al., 2018). The PD-1 receptors on activated T cells regulates the immune system during various physiological responses, including the responses to cancer and autoimmune diseases (LaFleur et al., 2018): in particular, the PD-L1/PD-1 axis in a tumor microenvironment is hijacked by cancer cells seeking to avoid immune surveillance (Li et al., 2018).

### α9-Mediated Signaling Pathways in Melanoma

Nguyen et al. (2019) used RT-PCR and Western blotting to analyze the expression of nAChRs and found that the expression of α9 nAChR mRNA was higher that of the other nAChR subunits in three melanoma cell lines (A375, A2058, and MDA-MB-435) and a primary melanocyte cell line (HEMn-LP), and that it was higher in melanoma cells than in melanocytes. They also found correlations between α9 mRNA expression and the clinical parameters of melanoma patients, and that high levels of α9 expression are significantly associated with lymph node metastases.

The *in vitro* over-expression of α9 in MDA-MB-435 and A2058 melanoma cells increased cell proliferation and migration, and this was paralleled by the activation of the Akt and ERK signaling pathways, and up-regulated PD-L1 expression. Furthermore, the suppression of α9 expression significantly inhibited melanoma cell growth and migration, decreased Akt and ERK activation, and reduced PD-L1 expression.

The over-expression of α9 nAChRs in A2058 and MDA-MB 435 cells also induced mesenchymal-like melanoma cells with the loss of cell-cell adhesion junctions, whereas α9 knockdown in A2058 cells led to an increase in the number of cell-cell adhesion junctions and epithelial-like melanoma cells.

A number of recent studies have shown positive correlations between smoking and PD-L1 expression: Wang et al. (2019) have shown that tobacco smoke induces PD-L1 expression in lung epithelial cells; and Nguyen et al. (2019) have found that nicotine induces α9 nAChR activity promoting melanoma cell proliferation in a dose- and time-dependent manner by stimulating the α9-mediated Akt, ERK, and STAT3 signaling pathways. In addition, nicotine-induced α9 nAChR activity up-regulated PD-L1 expression by activating the binding of transcription factor STAT3 to the PD-L1 promoter gene, thus suggesting that the functional activation of α9 nAChR by nicotine may regulate cancer-related immunity.

## Conclusions and Perspectives

The data described in this review clearly indicate that α9-containing nAChRs play an important role in various cancers. They are expressed in cell types other than neurons and can trigger ion fluxes and/or metabotropic pathways at different time scales. These receptors are higly permeable to Ca^2+^ and Thompson and Sontheimer (2019) have indirectly shown that α9 activation increases Ca^2+^ in cancer cells. This suggests that α9 receptors modulate various fundamental processes in signal transduction, which makes them powerful regulators of responses that stimulate a number of cancer processes, including cell proliferation, the spread of metastases, and the inhibition of apoptosis. This has been clearly established by the work of Lin et al. (2019), who used multiple RNA-seq and microarray datasets to show that α9-interacting proteins and signaling pathways are common in various cancers.

Many studies have shown the involvement of α7 nAChRs in tumor-related processes by using the antagonists αBgtx and MLA, which are also active on α9 nAChRs. It is therefore possible that the latter are involved in many more tumor processes than initially hypothesized.

Various clinical studies have shown that the expression of α9 subunits correlates with the unfavorable prognosis of cancer patients, and so α9 nAChRs may act as a novel biomarker when assessing risk and evaluating treatment options.

Greater understanding of the physiological role of α9-containing nAChRs in cells and the molecular mechanisms underlying the way in which they regulate the development of cancer will advance our knowledge of the biology and treatment of different cancers, and recognising their importance in the various stages of tumor progression may allow the design of effective anti-cancer drugs and therefore provide more and better options for cancer patients.

## Author Contributions

SP: conceptualization, conducting the research and investigation process, writing—review and editing. MZ: conceptualization, preparation, creation of the published work, writing—review and editing. FC: conceptualization, supervision, and funding acquisition. CG: conceptualization, supervision, statistical analysis, preparation, creation of the published work, writing—review and editing, and funding acquisition. All authors contributed to the article and approved the submitted version.

## Conflict of Interest

The authors declare that the research was conducted in the absence of any commercial or financial relationships that could be construed as a potential conflict of interest.

## Publisher’s Note

All claims expressed in this article are solely those of the authors and do not necessarily represent those of their affiliated organizations, or those of the publisher, the editors and the reviewers. Any product that may be evaluated in this article, or claim that may be made by its manufacturer, is not guaranteed or endorsed by the publisher.

## Footnotes

^1^ https://caintegrator.nci.nih.gov/rembrandt/

## References

[B1] Afrashteh Nour M. Hajiasgharzadeh K. Kheradmand F. Asadzadeh Z. Bolandi N. Baradaran B. (2021). Nicotinic acetylcholine receptors in chemotherapeutic drugs resistance: an emerging targeting candidate. Life Sci. 278:119557. 10.1016/j.lfs.2021.119557 33930371

[B2] Albuquerque E. X. Pereira E. F. Alkondon M. Rogers S. W. (2009). Mammalian nicotinic acetylcholine receptors: from structure to function. Physiol. Rev. 89, 73–120. 10.1152/physrev.00015.2008 19126755 PMC2713585

[B3] Banelli B. Carra E. Barbieri F. Wurth R. Parodi F. Pattarozzi A.. (2015). The histone demethylase KDM5A is a key factor for the resistance to temozolomide in glioblastoma. Cell Cycle 14, 3418–3429. 10.1080/15384101.2015.1090063 26566863 PMC4825557

[B4] Baumann L. Kauschke V. Vikman A. Durselen L. Krasteva-Christ G. Kampschulte M.. (2019). Deletion of nicotinic acetylcholine receptor alpha9 in mice resulted in altered bone structure. Bone 120, 285–296. 10.1016/j.bone.2018.11.003 30414510 PMC6492625

[B5] Bavo F. Pucci S. Fasoli F. Lammi C. Moretti M. Mucchietto V.. (2018). Potent antiglioblastoma agents by hybridizing the onium-alkyloxy-stilbene based structures of an alpha7-nAChR, alpha9-nAChR antagonist and of a pro-oxidant mitocan. J. Med. Chem. 61, 10531–10544. 10.1021/acs.jmedchem.8b01052 30403486

[B6] Boffi J. C. Marcovich I. Gill-Thind J. K. Corradi J. Collins T. Lipovsek M. M.. (2017). Differential contribution of subunit interfaces to alpha9alpha10 nicotinic acetylcholine receptor function. Mol. Pharmacol. 91, 250–262. 10.1124/mol.116.107482 28069778 PMC5325082

[B7] Borroni V. Barrantes F. J. (2021). Homomeric and heteromeric alpha7 nicotinic acetylcholine receptors in health and some central nervous system diseases. Membranes (Basel) 11:664. 10.3390/membranes11090664 34564481 PMC8465519

[B8] Bychkov M. Shenkarev Z. Shulepko M. Shlepova O. Kirpichnikov M. Lyukmanova E. (2019). Water-soluble variant of human Lynx1 induces cell cycle arrest and apoptosis in lung cancer cells *via* modulation of alpha7 nicotinic acetylcholine receptors. PLoS One 14:e0217339. 10.1371/journal.pone.0217339 31150435 PMC6544245

[B10] Chen J. Cheuk I. W. Y. Shin V. Y. Kwong A. (2019). Acetylcholine receptors: Key players in cancer development. Surg. Oncol. 31, 46–53. 10.1016/j.suronc.2019.09.003 31536927

[B9] Chen C. S. Lee C. H. Hsieh C. D. Ho C. T. Pan M. H. Huang C. S.. (2011). Nicotine-induced human breast cancer cell proliferation attenuated by garcinol through down-regulation of the nicotinic receptor and cyclin D3 proteins. Breast Cancer Res. Treat. 125, 73–87. 10.1007/s10549-010-0821-3 20229177

[B11] Chernyavsky A. I. Arredondo J. Vetter D. E. Grando S. A. (2007). Central role of alpha9 acetylcholine receptor in coordinating keratinocyte adhesion and motility at the initiation of epithelialization. Exp. Cell Res. 313, 3542–3555. 10.1016/j.yexcr.2007.07.011 17706194 PMC2682983

[B12] Chikova A. Bernard H. U. Shchepotin I. B. Grando S. A. (2012). New associations of the genetic polymorphisms in nicotinic receptor genes with the risk of lung cancer. Life Sci. 91, 1103–1108. 10.1016/j.lfs.2011.12.023 22280835 PMC3341501

[B13] Colomer C. Olivos-Ore L. A. Vincent A. Mcintosh J. M. Artalejo A. R. Guerineau N. C. (2010). Functional characterization of alpha9-containing cholinergic nicotinic receptors in the rat adrenal medulla: implication in stress-induced functional plasticity. J. Neurosci. 30, 6732–6742. 10.1523/JNEUROSCI.4997-09.2010 20463235 PMC2994257

[B14] Corradi J. Bouzat C. (2016). Understanding the bases of function and modulation of alpha7 nicotinic receptors: implications for drug discovery. Mol. Pharmacol. 90, 288–299. 10.1124/mol.116.104240 27190210

[B15] Corringer P. J. Poitevin F. Prevost M. S. Sauguet L. Delarue M. Changeux J. P. (2012). Structure and pharmacology of pentameric receptor channels: from bacteria to brain. Structure 20, 941–956. 10.1016/j.str.2012.05.003 22681900

[B16] Couturier S. Bertrand D. Matter J. M. Hernandez M. C. Bertrand S. Millar N.. (1990). A neuronal nicotinic acetylcholine receptor subunit (alpha 7) is developmentally regulated and forms a homo-oligomeric channel blocked by alpha-BTX. Neuron 5, 847–856. 10.1016/0896-6273(90)90344-f 1702646

[B17] Crespi A. Colombo S. F. Gotti C. (2018). Proteins and chemical chaperones involved in neuronal nicotinic receptor expression and function: an update. Br. J. Pharmacol. 175, 1869–1879. 10.1111/bph.13777 28294298 PMC5978959

[B18] Davies A. R. Hardick D. J. Blagbrough I. S. Potter B. V. Wolstenholme A. J. Wonnacott S. (1999). Characterisation of the binding of [3H]methyllycaconitine: a new radioligand for labelling alpha 7-type neuronal nicotinic acetylcholine receptors. Neuropharmacology 38, 679–690. 10.1016/s0028-3908(98)00221-4 10340305

[B19] Dobes M. Khurana V. G. Shadbolt B. Jain S. Smith S. F. Smee R.. (2011). Increasing incidence of glioblastoma multiforme and meningioma and decreasing incidence of schwannoma (2000–2008): findings of a multicenter australian study. Surg. Neurol. Int. 2:176. 10.4103/2152-7806.90696 22276231 PMC3263004

[B21] Elgoyhen A. B. Johnson D. S. Boulter J. Vetter D. E. Heinemann S. (1994). Alpha 9: an acetylcholine receptor with novel pharmacological properties expressed in rat cochlear hair cells. Cell 79, 705–715. 10.1016/0092-8674(94)90555-x 7954834

[B20] Elgoyhen A. B. Katz E. (2012). The efferent medial olivocochlear-hair cell synapse. J. Physiol. Paris 106, 47–56. 10.1016/j.jphysparis.2011.06.001 21762779 PMC3213294

[B22] Elgoyhen A. B. Vetter D. E. Katz E. Rothlin C. V. Heinemann S. F. Boulter J. (2001). alpha10: a determinant of nicotinic cholinergic receptor function in mammalian vestibular and cochlear mechanosensory hair cells. Proc. Natl. Acad. Sci. U S A 98, 3501–3506. 10.1073/pnas.051622798 11248107 PMC30682

[B23] Ellison M. Haberlandt C. Gomez-Casati M. E. Watkins M. Elgoyhen A. B. Mcintosh J. M.. (2006). Alpha-RgIA: a novel conotoxin that specifically and potently blocks the alpha9alpha10 nAChR. Biochemistry 45, 1511–1517. 10.1021/bi0520129 16445293

[B24] Fararjeh A. S. Tu S. H. Chen L. C. Cheng T. C. Liu Y. R. Chang H. L.. (2019). Long-term exposure to extremely low-dose of nicotine and 4-(methylnitrosamino)-1–(3-pyridyl)-1-butanone (NNK) induce non-malignant breast epithelial cell transformation through activation of the a9-nicotinic acetylcholine receptor-mediated signaling pathway. Environ. Toxicol. 34, 73–82. 10.1002/tox.22659 30259641

[B25] Ferlay J. Colombet M. Soerjomataram I. Parkin D. M. Pineros M. Znaor A.. (2021). Cancer statistics for the year 2020: an overview. Int. J. Cancer [Online ahead of print]. 10.1002/ijc.33588 33818764

[B26] Friedman J. R. Richbart S. D. Merritt J. C. Brown K. C. Nolan N. A. Akers A. T.. (2019). Acetylcholine signaling system in progression of lung cancers. Pharmacol. Ther. 194, 222–254. 10.1016/j.pharmthera.2018.10.002 30291908 PMC6348061

[B27] Fu X. W. Song P. F. Spindel E. R. (2015). Role of Lynx1 and related Ly6 proteins as modulators of cholinergic signaling in normal and neoplastic bronchial epithelium. Int. Immunopharmacol. 29, 93–98. 10.1016/j.intimp.2015.05.022 26025503 PMC4758445

[B28] Fucile S. Renzi M. Lax P. Eusebi F. (2003). Fractional Ca(2+) current through human neuronal alpha7 nicotinic acetylcholine receptors. Cell Calcium 34, 205–209. 10.1016/s0143-4160(03)00071-x 12810063

[B29] Fucile S. Sucapane A. Eusebi F. (2006). Ca2+ permeability through rat cloned alpha9-containing nicotinic acetylcholine receptors. Cell Calcium 39, 349–355. 10.1016/j.ceca.2005.12.002 16451809

[B30] Fujii T. Mashimo M. Moriwaki Y. Misawa H. Ono S. Horiguchi K.. (2017). Expression and function of the cholinergic system in immune cells. Front. Immunol. 8:1085. 10.3389/fimmu.2017.01085 28932225 PMC5592202

[B31] Gershenwald J. E. Guy G. P. Jr. (2015). Stemming the rising incidence of melanoma: calling prevention to action. J. Natl. Cancer Inst. 108:djv381. 10.1093/jnci/djv381 26563358 PMC6048594

[B32] Gotti C. Balestra B. Moretti M. Rovati G. E. Maggi L. Rossoni G.. (1998). 4-Oxystilbene compounds are selective ligands for neuronal nicotinic alphaBungarotoxin receptors. Br. J. Pharmacol. 124, 1197–1206. 10.1038/sj.bjp.0701957 9720791 PMC1565512

[B33] Gotti C. Zoli M. Clementi F. (2006). Brain nicotinic acetylcholine receptors: native subtypes and their relevance. Trends Pharmacol. Sci. 27, 482–491. 10.1016/j.tips.2006.07.004 16876883

[B34] Grando S. A. (2014). Connections of nicotine to cancer. Nat. Rev. Cancer 14, 419–429. 10.1038/nrc3725 24827506

[B35] Gu S. Knowland D. Matta J. A. O’carroll M. L. Davini W. B. Dhara M.. (2020). Hair cell alpha9alpha10 nicotinic acetylcholine receptor functional expression regulated by ligand binding and deafness gene products. Proc. Natl. Acad. Sci. U S A 117, 24534–24544. 10.1073/pnas.2013762117 32929005 PMC7533656

[B36] Guha P. Bandyopadhyaya G. Polumuri S. K. Chumsri S. Gade P. Kalvakolanu D. V.. (2014). Nicotine promotes apoptosis resistance of breast cancer cells and enrichment of side population cells with cancer stem cell-like properties *via* a signaling cascade involving galectin-3, alpha9 nicotinic acetylcholine receptor and STAT3. Breast Cancer Res. Treat. 145, 5–22. 10.1007/s10549-014-2912-z 24668500 PMC4028025

[B37] Hecker A. Kullmar M. Wilker S. Richter K. Zakrzewicz A. Atanasova S.. (2015). Phosphocholine-modified macromolecules and canonical nicotinic agonists inhibit ATP-induced IL-1beta release. J. Immunol. 195, 2325–2334. 10.4049/jimmunol.1400974 26202987

[B38] Hone A. J. Servent D. Mcintosh J. M. (2018). alpha9-containing nicotinic acetylcholine receptors and the modulation of pain. Br. J. Pharmacol. 175, 1915–1927. 10.1111/bph.13931 28662295 PMC5980226

[B39] Hou L. Jiang J. Liu B. Han W. Wu Y. Zou X.. (2016). Smoking and adult glioma: a population-based case-control study in China. Neuro Oncol. 18, 105–113. 10.1093/neuonc/nov146 26409568 PMC4677414

[B40] Hsieh Y. C. Lee C. H. Tu S. H. Wu C. H. Hung C. S. Hsieh M. C.. (2014). CHRNA9 polymorphisms and smoking exposure synergize to increase the risk of breast cancer in Taiwan. Carcinogenesis 35, 2520–2525. 10.1093/carcin/bgu179 25142973

[B41] Huang L. C. Lin C. L. Qiu J. Z. Lin C. Y. Hsu K. W. Tam K. W.. (2017). Nicotinic acetylcholine receptor subtype Alpha-9 mediates triple-negative breast cancers based on a spontaneous pulmonary metastasis mouse model. Front. Cell Neurosci. 11:336. 10.3389/fncel.2017.00336 29163048 PMC5675882

[B42] Hurst R. Rollema H. Bertrand D. (2013). Nicotinic acetylcholine receptors: from basic science to therapeutics. Pharmacol. Ther. 137, 22–54. 10.1016/j.pharmthera.2012.08.012 22925690

[B43] Improgo M. R. Schlichting N. A. Cortes R. Y. Zhao-Shea R. Tapper A. R. Gardner P. D. (2010). ASCL1 regulates the expression of the CHRNA5/A3/B4 lung cancer susceptibility locus. Mol. Cancer Res. 8, 194–203. 10.1158/1541-7786.MCR-09-0185 20124469 PMC2824774

[B44] Improgo M. R. Soll L. G. Tapper A. R. Gardner P. D. (2013). Nicotinic acetylcholine receptors mediate lung cancer growth. Front. Physiol. 4:251. 10.3389/fphys.2013.00251 24062692 PMC3774984

[B45] Indurthi D. C. Pera E. Kim H. L. Chu C. Mcleod M. D. Mcintosh J. M.. (2014). Presence of multiple binding sites on alpha9alpha10 nAChR receptors alludes to stoichiometric-dependent action of the alpha-conotoxin, Vc1.1. Biochem. Pharmacol. 89, 131–140. 10.1016/j.bcp.2014.02.002 24548457 PMC5089836

[B46] Jemal A. Bray F. Center M. M. Ferlay J. Ward E. Forman D. (2011). Global cancer statistics. CA Cancer J. Clin. 61, 69–90. 10.3322/caac.20107 21296855

[B48] Jones M. S. Jones P. C. Stern S. L. Elashoff D. Hoon D. S. B. Thompson J.. (2017). The impact of smoking on sentinel node metastasis of primary cutaneous melanoma. Ann. Surg. Oncol. 24, 2089–2094. 10.1245/s10434-017-5775-9 28224364 PMC5553293

[B49] Kabbani N. Nichols R. A. (2018). Beyond the channel: metabotropic signaling by nicotinic receptors. Trends Pharmacol. Sci. 39, 354–366. 10.1016/j.tips.2018.01.002 29428175

[B50] Khalil A. A. Jameson M. J. Broaddus W. C. Lin P. S. Chung T. D. (2013). Nicotine enhances proliferation, migration and radioresistance of human malignant glioma cells through EGFR activation. Brain Tumor Pathol. 30, 73–83. 10.1007/s10014-012-0101-5 22614999

[B51] King J. R. Kabbani N. (2016). Alpha 7 nicotinic receptor coupling to heterotrimeric G proteins modulates RhoA activation, cytoskeletal motility and structural growth. J. Neurochem. 138, 532–545. 10.1111/jnc.13660 27167578

[B52] King J. R. Nordman J. C. Bridges S. P. Lin M. K. Kabbani N. (2015). Identification and characterization of a G protein-binding cluster in alpha7 nicotinic acetylcholine receptors. J. Biol. Chem. 290, 20060–20070. 10.1074/jbc.M115.647040 26088141 PMC4536413

[B53] Kryukova E. V. Egorova N. S. Kudryavtsev D. S. Lebedev D. S. Spirova E. N. Zhmak M. N.. (2019). From synthetic fragments of endogenous three-finger proteins to potential drugs. Front. Pharmacol. 10:748. 10.3389/fphar.2019.00748 31333465 PMC6616073

[B54] LaFleur M. W. Muroyama Y. Drake C. G. Sharpe A. H. (2018). Inhibitors of the PD-1 pathway in tumor therapy. J. Immunol. 200, 375–383. 10.4049/jimmunol.1701044 29311378 PMC5924692

[B55] Lau J. K. Brown K. C. Thornhill B. A. Crabtree C. M. Dom A. M. Witte T. R.. (2013). Inhibition of cholinergic signaling causes apoptosis in human bronchioalveolar carcinoma. Cancer Res. 73, 1328–1339. 10.1158/0008-5472.CAN-12-3190 23222296 PMC10461321

[B56] Lee C. H. Chang Y. C. Chen C. S. Tu S. H. Wang Y. J. Chen L. C.. (2011). Crosstalk between nicotine and estrogen-induced estrogen receptor activation induces alpha9-nicotinic acetylcholine receptor expression in human breast cancer cells. Breast Cancer Res. Treat. 129, 331–345. 10.1007/s10549-010-1209-0 20953833

[B57] Lee C. H. Huang C. S. Chen C. S. Tu S. H. Wang Y. J. Chang Y. J.. (2010). Overexpression and activation of the alpha9-nicotinic receptor during tumorigenesis in human breast epithelial cells. J. Natl. Cancer Inst. 102, 1322–1335. 10.1093/jnci/djq300 20733118

[B58] Lee K. L. Kuo Y. C. Ho Y. S. Huang Y. H. (2019). Triple-negative breast cancer: current understanding and future therapeutic breakthrough targeting cancer stemness. Cancers (Basel) 11:1334. 10.3390/cancers11091334 31505803 PMC6769912

[B59] Li H. X. Peng X. X. Zong Q. Zhang K. Wang M. X. Liu Y. Z.. (2016). Cigarette smoking and risk of adult glioma: a meta-analysis of 24 observational studies involving more than 2.3 million individuals. Onco Targets Ther. 9, 3511–3523. 10.2147/OTT.S99713 27366088 PMC4913539

[B60] Li X. Shao C. Shi Y. Han W. (2018). Lessons learned from the blockade of immune checkpoints in cancer immunotherapy. J. Hematol. Oncol. 11:31. 10.1186/s13045-018-0578-4 29482595 PMC6389077

[B61] Li X. Tae H. S. Chu Y. Jiang T. Adams D. J. Yu R. (2021). Medicinal chemistry, pharmacology and therapeutic potential of alpha-conotoxins antagonizing the alpha9alpha10 nicotinic acetylcholine receptor. Pharmacol. Ther. 222:107792. 10.1016/j.pharmthera.2020.107792 33309557

[B62] Liang J. Tae H.-S. Xu X. Jiang T. Adams D. J. Yu R. (2020). Dimerization of alpha-conotoxins as a strategy to enhance the inhibition of the human α7 and α9α10 nicotinic acetylcholine receptors. J. Med. Chem. 63, 2974–2985. 10.1021/acs.jmedchem.9b01536 32101438

[B63] Lin C. Y. Lee C. H. Chuang Y. H. Lee J. Y. Chiu Y. Y. Wu Lee Y. H.. (2019). Membrane protein-regulated networks across human cancers. Nat. Commun. 10:3131. 10.1038/s41467-019-10920-8 31311925 PMC6635409

[B64] Liu Q. Li M. Whiteaker P. Shi F. D. Morley B. J. Lukas R. J. (2019). Attenuation in nicotinic acetylcholine receptor alpha9 and alpha10 subunit double knock-out mice of experimental autoimmune encephalomyelitis. Biomolecules 9:827. 10.3390/biom9120827 31817275 PMC6995583

[B65] Liu Q. Whiteaker P. Morley B. J. Shi F. D. Lukas R. J. (2017). Distinctive roles for alpha7*- and alpha9*-nicotinic acetylcholine receptors in inflammatory and autoimmune responses in the murine experimental autoimmune encephalomyelitis model of multiple sclerosis. Front. Cell Neurosci. 11:287. 10.3389/fncel.2017.00287 29018331 PMC5615207

[B66] Louis D. N. Holland E. C. Cairncross J. G. (2001). Glioma classification: a molecular reappraisal. Am. J. Pathol. 159, 779–786. 10.1016/S0002-9440(10)61750-6 11549567 PMC1850454

[B67] Luo S. Zhangsun D. Harvey P. J. Kaas Q. Wu Y. Zhu X.. (2015). Cloning, synthesis and characterization of alphaO-conotoxin GeXIVA, a potent alpha9alpha10 nicotinic acetylcholine receptor antagonist. Proc. Natl. Acad. Sci. U S A 112, E4026–E4035. 10.1073/pnas.1503617112 26170295 PMC4522777

[B68] Lustig L. R. Peng H. Hiel H. Yamamoto T. And Fuchs P. A. (2001). Molecular cloning and mapping of the human nicotinic acetylcholine receptor alpha10 (CHRNA10). Genomics 73, 272–283. 10.1006/geno.2000.6503 11350119

[B69] Marcovich I. Moglie M. J. Carpaneto Freixas A. E. Trigila A. P. Franchini L. F. Plazas P. V.. (2020). Distinct evolutionary trajectories of neuronal and hair cell nicotinic acetylcholine receptors. Mol. Biol. Evol. 37, 1070–1089. 10.1093/molbev/msz290 31821508 PMC7086180

[B70] Matta J. A. Gu S. Davini W. B. Bredt D. S. (2021). Nicotinic acetylcholine receptor redux: discovery of accessories opens therapeutic vistas. Science 373:eabg6539. 10.1126/science.abg6539 34385370

[B71] Mcintosh J. M. Plazas P. V. Watkins M. Gomez-Casati M. E. Olivera B. M. Elgoyhen A. B. (2005). A novel alpha-conotoxin, PeIA, cloned from conus pergrandis, discriminates between rat alpha9alpha10 and alpha7 nicotinic cholinergic receptors. J. Biol. Chem. 280, 30107–30112. 10.1074/jbc.M504102200 15983035

[B72] Mcneill J. Rudyk C. Hildebrand M. E. Salmaso N. (2021). Ion channels and electrophysiological properties of astrocytes: implications for emergent stimulation technologies. Front. Cell Neurosci. 15:644126. 10.3389/fncel.2021.644126 34093129 PMC8173131

[B73] Meza R. Meernik C. Jeon J. Cote M. L. (2015). Lung cancer incidence trends by gender, race and histology in the united states, 1973–2010. PLoS One 10:e0121323. 10.1371/journal.pone.0121323 25822850 PMC4379166

[B74] Miwa J. M. (2021). Lynx1 prototoxins: critical accessory proteins of neuronal nicotinic acetylcholine receptors. Curr. Opin. Pharmacol. 56, 46–51. 10.1016/j.coph.2020.09.016 33254061 PMC8771676

[B75] Morley B. J. Dolan D. F. Ohlemiller K. K. Simmons D. D. (2017). Generation and characterization of alpha9 and alpha10 nicotinic acetylcholine receptor subunit knockout mice on a C57BL/6J background. Front. Neurosci. 11:516. 10.3389/fnins.2017.00516 28983232 PMC5613126

[B76] Morley B. J. Whiteaker P. Elgoyhen A. B. (2018). Commentary: nicotinic acetylcholine receptor alpha9 and alpha10 subunits are expressed in the brain of mice. Front. Cell Neurosci. 12:104. 10.3389/fncel.2018.00104 29765305 PMC5938352

[B77] Mucchietto V. Crespi A. Fasoli F. Clementi F. Gotti C. (2016). Neuronal acetylcholine nicotinic receptors as new targets for lung cancer treatment. Curr. Pharm. Des. 22, 2160–2169. 10.2174/1381612822666160203144114 26845123

[B78] Mucchietto V. Fasoli F. Pucci S. Moretti M. Benfante R. Maroli A.. (2018). alpha9- and alpha7-containing receptors mediate the pro-proliferative effects of nicotine in the A549 adenocarcinoma cell line. Br. J. Pharmacol. 175, 1957–1972. 10.1111/bph.13954 28726253 PMC5980168

[B79] Nguyen H. D. Liao Y. C. Ho Y. S. Chen L. C. Chang H. W. Cheng T. C.. (2019). The alpha9 nicotinic acetylcholine receptor mediates nicotine-induced PD-L1 expression and regulates melanoma cell proliferation and migration. Cancers (Basel) 11:1991. 10.3390/cancers11121991 31835799 PMC6966517

[B80] Nguyen V. T. Ndoye A. Grando S. A. (2000). Novel human alpha9 acetylcholine receptor regulating keratinocyte adhesion is targeted by Pemphigus vulgaris autoimmunity. Am. J. Pathol. 157, 1377–1391. 10.1016/s0002-9440(10)64651-2 11021840 PMC1850172

[B81] Nordman J. C. Kabbani N. (2014). Microtubule dynamics at the growth cone are mediated by alpha7 nicotinic receptor activation of a Galphaq and IP3 receptor pathway. FASEB J. 28, 2995–3006. 10.1096/fj.14-251439 24687992 PMC4062821

[B82] Ostrom Q. T. Gittleman H. Fulop J. Liu M. Blanda R. Kromer C.. (2015). CBTRUS statistical report: primary brain and central nervous system tumors diagnosed in the united states in 2008–2012. Neuro Oncol. 17, iv1–iv62. 10.1093/neuonc/nov189 26511214 PMC4623240

[B83] Papke R. L. Lindstrom J. M. (2020). Nicotinic acetylcholine receptors: conventional and unconventional ligands and signaling. Neuropharmacology 168:108021. 10.1016/j.neuropharm.2020.108021 32146229 PMC7610230

[B84] Pesch B. Kendzia B. Gustavsson P. Jockel K. H. Johnen G. Pohlabeln H.. (2012). Cigarette smoking and lung cancer–relative risk estimates for the major histological types from a pooled analysis of case-control studies. Int. J. Cancer 131, 1210–1219. 10.1002/ijc.27339 22052329 PMC3296911

[B85] Plazas P. V. Katz E. Gomez-Casati M. E. Bouzat C. Elgoyhen A. B. (2005). Stoichiometry of the alpha9alpha10 nicotinic cholinergic receptor. J. Neurosci. 25, 10905–10912. 10.1523/JNEUROSCI.3805-05.2005 16306403 PMC6725887

[B86] Pucci S. Bolchi C. Bavo F. Pallavicini M. De Palma C. Renzi M.. (2022). Evidence of a dual mechanism of action underlying the anti-proliferative and cytotoxic effects of ammonium-alkyloxy-stilbene-based α7- and α9- nicotinic ligands on glioblastoma cells. Pharmacol. Res. 105959. 10.1016/j.phrs.2021.105959 34756924

[B87] Pucci S. Fasoli F. Moretti M. Benfante R. Di Lascio S. Viani P.. (2021). Choline and nicotine increase glioblastoma cell proliferation by binding and activating alpha7- and alpha9- containing nicotinic receptors. Pharmacol. Res. 163:105336. 10.1016/j.phrs.2020.105336 33276105

[B88] Richter K. Mathes V. Fronius M. Althaus M. Hecker A. Krasteva-Christ G.. (2016). Phosphocholine - an agonist of metabotropic but not of ionotropic functions of alpha9-containing nicotinic acetylcholine receptors. Sci. Rep. 6:28660. 10.1038/srep28660 27349288 PMC4923896

[B89] Romero H. K. Christensen S. B. Di Cesare Mannelli L. Gajewiak J. Ramachandra R. Elmslie K. S.. (2017). Inhibition of alpha9alpha10 nicotinic acetylcholine receptors prevents chemotherapy-induced neuropathic pain. Proc. Natl. Acad. Sci. U S A 114, E1825–E1832. 10.1073/pnas.1621433114 28223528 PMC5347537

[B90] Salmaninejad A. Valilou S. F. Shabgah A. G. Aslani S. Alimardani M. Pasdar A.. (2019). PD-1/PD-L1 pathway: basic biology and role in cancer immunotherapy. J. Cell Physiol. 234, 16824–16837. 10.1002/jcp.28358 30784085

[B91] Samet J. M. Avila-Tang E. Boffetta P. Hannan L. M. Olivo-Marston S. Thun M. J.. (2009). Lung cancer in never smokers: clinical epidemiology and environmental risk factors. Clin. Cancer Res. 15, 5626–5645. 10.1158/1078-0432.CCR-09-0376 19755391 PMC3170525

[B92] Schaal C. Chellappan S. P. (2014). Nicotine-mediated cell proliferation and tumor progression in smoking-related cancers. Mol. Cancer Res. 12, 14–23. 10.1158/1541-7786.MCR-13-0541 24398389 PMC3915512

[B93] Schane R. E. Ling P. M. Glantz S. A. (2010). Health effects of light and intermittent smoking: a review. Circulation 121, 1518–1522. 10.1161/CIRCULATIONAHA.109.904235 20368531 PMC2865193

[B94] Schuller H. M. (2009). Is cancer triggered by altered signalling of nicotinic acetylcholine receptors. Nat. Rev. Cancer 9, 195–205. 10.1038/nrc2590 19194381

[B95] Sgard F. Charpantier E. Bertrand S. Walker N. Caput D. Graham D.. (2002). A novel human nicotinic receptor subunit, alpha10, that confers functionality to the alpha9-subunit. Mol. Pharmacol. 61, 150–159. 10.1124/mol.61.1.150 11752216

[B96] Shao C. Zhao W. Qi Z. He J. (2016). Smoking and glioma risk: evidence from a meta-analysis of 25 observational studies. Medicine (Baltimore) 95:e2447. 10.1097/MD.0000000000002447 26765433 PMC4718259

[B97] Sharma G. Vijayaraghavan S. (2001). Nicotinic cholinergic signaling in hippocampal astrocytes involves calcium-induced calcium release from intracellular stores. Proc. Natl. Acad. Sci. U S A 98, 4148–4153. 10.1073/pnas.071540198 11259680 PMC31194

[B98] Shaw H. M. Milton G. W. (1981). Smoking and the development of metastases from malignant melanoma. Int. J. Cancer 28, 153–156. 10.1002/ijc.2910280207 7319670

[B99] Slattery M. L. Curtin K. Giuliano A. R. Sweeney C. Baumgartner R. Edwards S.. (2008). Active and passive smoking, IL6, ESR1 and breast cancer risk. Breast Cancer Res. Treat. 109, 101–111. 10.1007/s10549-007-9629-1 17594514 PMC2532584

[B100] Spina R. Voss D. M. Asnaghi L. Sloan A. Bar E. E. (2016). Atracurium Besylate and other neuromuscular blocking agents promote astroglial differentiation and deplete glioblastoma stem cells. Oncotarget 7, 459–472. 10.18632/oncotarget.6314 26575950 PMC4808011

[B101] Spindel E. R. (2016). Cholinergic targets in lung cancer. Curr. Pharm. Des. 22, 2152–2159. 10.2174/1381612822666160127114237 26818857 PMC4961355

[B102] Sun Z. Bao J. Zhangsun M. Dong S. Zhangsun D. Luo S. (2020). alphaO-conotoxin GeXIVA inhibits the growth of breast cancer cells *via* interaction with alpha9 nicotine acetylcholine receptors. Mar. Drugs 18:195. 10.3390/md18040195 32272701 PMC7231225

[B103] Tang M. S. Wu X. R. Lee H. W. Xia Y. Deng F. M. Moreira A. L.. (2019). Electronic-cigarette smoke induces lung adenocarcinoma and bladder urothelial hyperplasia in mice. Proc. Natl. Acad. Sci. U S A 116, 21727–21731. 10.1073/pnas.1911321116 31591243 PMC6815158

[B104] Thompson E. G. Sontheimer H. (2019). Acetylcholine receptor activation as a modulator of glioblastoma invasion. Cells 8:1203. 10.3390/cells8101203 31590360 PMC6829263

[B105] Treinin M. Papke R. L. Nizri E. Ben-David Y. Mizrachi T. Brenner T. (2017). Role of the alpha7 nicotinic acetylcholine receptor and RIC-3 in the cholinergic anti-inflammatory pathway. Cent. Nerv. Syst. Agents Med. Chem. 17, 90–99. 10.2174/1871524916666160829114533 27573666

[B106] Tu S. H. Lin Y. C. Huang C. C. Yang P. S. Chang H. W. Chang C. H.. (2016). Protein phosphatase Mg2+/Mn2+ dependent 1F promotes smoking-induced breast cancer by inactivating phosphorylated-p53-induced signals. Oncotarget 7, 77516–77531. 10.18632/oncotarget.12717 27769050 PMC5363601

[B107] Verbitsky M. Rothlin C. V. Katz E. Elgoyhen A. B. (2000). Mixed nicotinic-muscarinic properties of the alpha9 nicotinic cholinergic receptor. Neuropharmacology 39, 2515–2524. 10.1016/s0028-3908(00)00124-6 11044723

[B108] Vetter D. E. Katz E. Maison S. F. Taranda J. Turcan S. Ballestero J.. (2007). The alpha10 nicotinic acetylcholine receptor subunit is required for normal synaptic function and integrity of the olivocochlear system. Proc. Natl. Acad. Sci. U S A 104, 20594–20599. 10.1073/pnas.0708545105 18077337 PMC2154476

[B109] Vetter D. E. Liberman M. C. Mann J. Barhanin J. Boulter J. Brown M. C.. (1999). Role of alpha9 nicotinic ACh receptor subunits in the development and function of cochlear efferent innervation. Neuron 23, 93–103. 10.1016/s0896-6273(00)80756-4 10402196

[B111] Wang Y. Sanghvi M. Gribizis A. Zhang Y. Song L. Morley B.. (2021). Efferent feedback controls bilateral auditory spontaneous activity. Nat. Commun. 12:2449. 10.1038/s41467-021-22796-8 33907194 PMC8079389

[B110] Wang G. Z. Zhang L. Zhao X. C. Gao S. H. Qu L. W. Yu H.. (2019). The Aryl hydrocarbon receptor mediates tobacco-induced PD-L1 expression and is associated with response to immunotherapy. Nat. Commun. 10:1125. 10.1038/s41467-019-08887-7 30850589 PMC6408580

[B112] Wessler I. Kirkpatrick C. J. (2008). Acetylcholine beyond neurons: the non-neuronal cholinergic system in humans. Br. J. Pharmacol. 154, 1558–1571. 10.1038/bjp.2008.185 18500366 PMC2518461

[B113] Yu R. Tae H. S. Tabassum N. Shi J. Jiang T. Adams D. J. (2018). Molecular determinants conferring the stoichiometric-dependent activity of alpha-conotoxins at the human alpha9alpha10 nicotinic acetylcholine receptor subtype. J. Med. Chem. 61, 4628–4634. 10.1021/acs.jmedchem.8b00115 29733583

[B114] Zhang Q. Ganapathy S. Avraham H. Nishioka T. Chen C. (2020). Nicotine exposure potentiates lung tumorigenesis by perturbing cellular surveillance. Br. J. Cancer 122, 904–911. 10.1038/s41416-020-0730-0 32001831 PMC7078213

[B115] Zheng N. Christensen S. B. Dowell C. Purushottam L. Skalicky J. J. Mcintosh J. M.. (2021). Discovery of methylene thioacetal-incorporated alpha-RgIA analogues as potent and stable antagonists of the human alpha9alpha10 nicotinic acetylcholine receptor for the treatment of neuropathic pain. J. Med. Chem. 64, 9513–9524. 10.1021/acs.jmedchem.1c00802 34161094 PMC8734577

[B116] Zhu P. Du X. L. Lu G. Zhu J. J. (2017). Survival benefit of glioblastoma patients after FDA approval of temozolomide concomitant with radiation and bevacizumab: a population-based study. Oncotarget 8, 44015–44031. 10.18632/oncotarget.17054 28467795 PMC5546458

[B117] Zoli M. Moretti M. Zanardi A. Mcintosh J. M. Clementi F. Gotti C. (2002). Identification of the nicotinic receptor subtypes expressed on dopaminergic terminals in the rat striatum. J. Neurosci. 22, 8785–8789. 10.1523/JNEUROSCI.22-20-08785.2002 12388584 PMC6757689

[B118] Zoli M. Pistillo F. Gotti C. (2015). Diversity of native nicotinic receptor subtypes in mammalian brain. Neuropharmacology 96, 302–311. 10.1016/j.neuropharm.2014.11.003 25460185

[B119] Zoli M. Pucci S. Vilella A. Gotti C. (2018). Neuronal and extraneuronal nicotinic acetylcholine receptors. Curr. Neuropharmacol. 16, 338–349. 10.2174/1570159X15666170912110450 28901280 PMC6018187

[B120] Zouridakis M. Giastas P. Zarkadas E. Chroni-Tzartou D. Bregestovski P. Tzartos S. J. (2014). Crystal structures of free and antagonist-bound states of human alpha9 nicotinic receptor extracellular domain. Nat. Struct. Mol. Biol. 21, 976–980. 10.1038/nsmb.2900 25282151

[B121] Zouridakis M. Papakyriakou A. Ivanov I. A. Kasheverov I. E. Tsetlin V. Tzartos S.. (2019). Crystal structure of the monomeric extracellular domain of alpha9 nicotinic receptor subunit in complex with alpha-conotoxin RgIA: molecular dynamics insights into RgIA binding to alpha9alpha10 nicotinic receptors. Front. Pharmacol. 10:474. 10.3389/fphar.2019.00474 31118896 PMC6504684

