# Supplementary material for: α9-Containing Nicotinic Receptors in Cancer
Source: Front Cell Neurosci. 2022 Jan 21;15:805123. doi: 10.3389/fncel.2021.805123 (PMC8814915; doi:10.3389/fncel.2021.805123)
Supplement: Supplementary file 1 [file Table_1.DOCX]

**Supplementary Table 1: Expression of α9 and α10 nAChR mRNA and/or protein in the indicated human cancer cells or tissue types.**

| **Cancer type / site** | **Cell / tissue type** | **α9 nAChR subunit** | **α10 nAChR subunit** | ***References*** |
| --- | --- | --- | --- | --- |
| **Lung**  (squamous cell carcinoma) | Patient samples, NCI-H157, NCI-H1703, NCI-H520,  NCI-H1869, NCI-H2170,  SW900, NCI-H1975 | mRNA  protein (WB, IF) | mRNA | *West et al., 2003; Tsurutami et al., 2005;*  *Improgo et al., 2010; Lee et al., 2010;*  *Chernyavsky et al., 2015; Mucchietto et al., 2018* |
| **Lung**  (adenocarcinoma) | A-549, NCI-H23,  NCI-H2347, HKULC 4,  NCI-H1395 | mRNA  protein (WB, IF) | mRNA | *Tsurutami et al., 2005; Dasgupta et al., 2006;*  *Lam et al, 2007; Improgo et al., 2010;*  *Mucchietto et al., 2018; Lin et al., 2019;*  *Qian et al., 2019* |
| **Lung**  (metastatic large cell carcinoma from lymph node) | NCI-H1299 | mRNA  protein (WB) | mRNA | *Dasgupta et al., 2006; Qian et al., 2019* |
| **Lung**  (metastatic adenocarcinoma from pleural effusion) | NCI-H1437, NCI-H2122,  HKULC 2, HKULC 3 | mRNA | mRNA | *Lam et al, 2007* |
| **Lung**  (metastatic adenocarcinoma from lymph node) | NCI-H1648, NCI-H1819,  NCI-H1993, NCI-H2009,  NCI-H2087, NCI-H838,  NCI-H1734, NCI-H1793 | mRNA | mRNA | *Lam et al, 2007* |
| **Lung**  (metastatic NSCC from lymph node) | NCI-H1770 | mRNA | mRNA | *Lam et al, 2007* |
| **Lung**  (metastatic adenocarcinoma from pericardial effusion) | HKULC 1 | mRNA | mRNA | *Lam et al, 2007* |
| **Lung**  (NSCC) | Patient samples, NCI-H1915 | mRNA | mRNA | *Lam et al., 2007* |
| **Lung**  (SCC) | Patient samples, DMS 53,  DMS-114 | mRNA  protein (WB) | mRNA | *Improgo et al., 2010; Qian et al., 2019* |
| **Lung**  (metastatic SCC from pleural effusion) | NCI-H69, NCI-H82,  NCI-H128 | mRNA | mRNA | *Improgo et al., 2010* |
| **Lung**  (metastatic SCC from bone marrow) | NCI-H146, NCI-H209 | mRNA | mRNA | *Improgo et al., 2010* |
| **Lung**  (metastatic SCC from pleural effusion) | NCI-H446, NCI-H460 | mRNA | mRNA | *Improgo et al., 2010* |
| **Lung**  (metastatic large cell carcinoma from lymph node) | NCI-H661 | mRNA | mRNA | *Improgo et al., 2010* |
| **Lung**  (large cell carcinoma) | NCI-H1581 | mRNA | mRNA | *Improgo et al., 2010* |
| **Lung**  (metastatic squamous cell carcinoma from pleural effusion) | SK-MES-1 | mRNA | mRNA | *Improgo et al., 2010* |
| **Lung**  (NNK- and HPV18-transformed bronchial epithelial cells) | NNK-transformed BEP2D (derived from BEP2D) | mRNA |  | *Chikova and Grando, 2011* |
| **Lung**  (metastatic SCC from liver) | NCI-H1688 | mRNA  protein (WB) | mRNA  protein (WB) | *Qian et al., 2019* |
| **Breast**  (metastatic from pleural effusion) | MCF-7, MDA-MB-157 | mRNA  protein (FACS, WB, GeXIVA) | mRNA  protein (FACS, WB, GeXIVA) | *Guo et al., 2008; Lee et al., 2010;*  *Chen et al., 2011; Tu et al., 2011;*  *Lee et al., 2011; Guha et al., 2014;*  *Kalantari-Dehaghi et al., 2015; Tu et al., 2016;*  *Sun et al., 2020a; Sun et al., 2020b* |
| **Breast**  (metastatic adenocarcinoma from pleural effusion) | MDA-MB-231, AU-565,  SK-BR-3, MDA-MB-468 | mRNA  protein (FACS, WB, IF, GeXIVA) | mRNA  protein (FACS, GeXIVA) | *Lee et al., 2010; Shih et al., 2010;*  *Chen et al., 2011; Tu et al., 2011;*  *Tu et al, 2016; Lin et al., 2019;*  *Sun et al., 2020a* |
| **Breast**  (metastatic adenocarcinoma from pericardial effusion) | MDA-MB-453 | mRNA  protein (FACS, WB, GeXIVA) | mRNA  protein (FACS, GeXIVA) | *Lee et al., 2010; Tu et al., 2011;*  *Tu et al., 2016; Sun et al., 2020a* |
| **Breast** | Patient sample, BT-483  BT-474, UACC-893  Hs 578T, BT-549  BT-20 | mRNA  protein (IHC, FACS, WB, IF, GeXIVA) | mRNA  protein (FACS, GeXIVA) | *Lee et al., 2010; Chen et al., 2011*  *Tu et al., 2011; Guha et al., 2014*  *Tu et al., 2016; Lin et al., 2019*  *Sun et al., 2020a* |
| **Breast**  (adenocarcinoma) | Patient samples | mRNA  protein (IHC) |  | *Lee et al., 2010* |
| **Breast**  (nicotine-transformed spontaneously immortalized mammary epithelial cells) | MCF-10A-Nic (derived from MCF-10A) | protein (WB) |  | *Chen et al., 2011* |
| **Breast**  (NNK-transformed spontaneously immortalized mammary epithelial cells) | NNK-transformed MCF-10A | mRNA |  | *Kalantari-Dehaghi et al., 2015* |
| **Breast**  (metastatic from pleural effusion) | T-47D, MDA-MB-436 | protein (WB) |  | *Tu et al., 2016; Lin et al., 2019* |
| **Breast**  (metastatic from ascites) | ZR-75-1, ZR-75-30 | mRNA  protein (FACS, WB, GeXIVA) | mRNA  protein (FACS, GeXIVA) | *Tu et al., 2016; Sun et al., 2020a* |
| **Breast**  (serial spontaneous metastatic adenocarcinoma from pleural effusion implanted in immune-competent SCID mice) | Highly metastatic TNBC (derived from MDA-MB-231 lung metastases) | mRNA  protein (WB) |  | *Huang et al., 2017* |
| **Breast**  (nicotine-, NNK- and SV40-transformed epithelial cells from milk) | Nicotine+NNK-chronically induced HBL-100 (derived from HBL-100) | protein (WB) |  | *Fararjeh et al., 2019* |
| **Breast**  (ductal carcinoma) | HCC1954, HCC1419,  HCC1937, HCC1395 | mRNA  protein (FACS, WB, GeXIVA) | mRNA  protein (FACS, GeXIVA) | *Lin et al., 2019; Sun et al., 2020a* |
| **Breast**  (from ascites) | Ehrlich-Lettre ascites strain E (mouse) | protein (RgIA) | protein (RgIA) | *Terpinskaya et al., 2020* |
| **Breast**  (metastatic adenocarcinoma from brain) | MDA-MB-361 | mRNA  protein (FACS, WB, GeXIVA) | mRNA  protein (FACS, GeXIVA) | *Sun et al., 2020a* |
| **Breast**  (acantholytic variant of squamous cell carcinoma) | HCC1806 | mRNA  protein (FACS, WB, GeXIVA) | mRNA  protein (FACS, GeXIVA) | *Sun et al., 2020a* |
| **Glioma** | Patient samples (REMBRANDT dataset) | mRNA |  | *Spina et al., 2016* |
| **Glioma**  (GBM) | Patient samples (TCGA dataset on Oncomine),  Patient samples (TCGA dataset via cBioPortal platform),  U-87MG ATCC, GBM5, A-172 | mRNA  protein (RgIA) | mRNA  protein (RgIA) | *Spina et al., 2016; Thompson and Sontheimer, 2020; Pucci et al., 2021a; Kolodziej et al., 2021* |
| **Glioma**  (primary GBM) | Patient samples,  Primary cell lines | mRNA |  | *Kolodziej et al., 2021* |
| **Glioma**  (recurrent GBM) | Patient samples,  Primary cell lines | mRNA |  | *Kolodziej et al., 2021* |
| **Glioma**  (gliosarcoma) | NCE-G 28 | mRNA | mRNA | *Kolodziej et al., 2021* |
| **Melanoma**  (amelanotic) | A-375 | mRNA  protein (WB) | mRNA | *Nguyen et al, 2019* |
| **Melanoma**  (metastatic amelanotic from lymph node) | A2058 | mRNA  protein (WB) | mRNA | *Nguyen et al, 2019* |
| **Melanoma**  (metastatic subcutaneous amelanotic from right buttock) | MDA-MB-435 | mRNA  protein (WB) | mRNA | *Nguyen et al, 2019* |
| **Melanoma**  (primary) | Patient samples (TCGA-SKCM cohort from UCSC Xena browser),  Cell lines (from R2 MegaSampler platform) | mRNA  protein (IHC) |  | *Nguyen et al, 2019* |
| **Melanoma**  (metastatic) | Patient samples (TCGA-SKCM cohort from UCSC Xena browser),  Cell lines (from R2 MegaSampler platform) | mRNA  protein (IHC) |  | *Nguyen et al, 2019* |

**WB**: Western blot ; **IF**: immunofluorescence ; **NSCC**: non-small-cell cancer ; **SCC**: small-cell cancer ; **NNK**: 4-(methylnitrosamino)-1-(3-pyridyl)-1-butanone ; **HPV**: human papilloma virus ; **FACS:** fluorescence-activated cell sorting ; **IHC**: immunohistochemistry ; **SCID**: severe combined immunodeficient ; **TNBC**: triple-negative breast cancer ; **SV40**: Simian virus 40 ; **REMBRANDT**: repository of molecular brain neoplasia data ; **GBM**: glioblastoma ; **TCGA**: the cancer genome atlas ; **SKCM**: skin cutaneous melanoma ; **UCSC**: University of California, Santa Cruz .

**References**

Chen, C.S., Lee, C.H., Hsieh, C.D., Ho, C.T., Pan, M.H., Huang, C.S., Tu, S.H., Wang, Y.J., Chen, L.C., Chang, Y.J., Wei, P.L., Yang, Y.Y., Wu, C.H., and Ho, Y.S. (2011). Nicotine-induced human breast cancer cell proliferation attenuated by garcinol through down-regulation of the nicotinic receptor and cyclin D3 proteins. *Breast Cancer Res Treat* 125**,** 73-87.

Chernyavsky, A.I., Shchepotin, I.B., Galitovkiy, V., and Grando, S.A. (2015). Mechanisms of tumor-promoting activities of nicotine in lung cancer: synergistic effects of cell membrane and mitochondrial nicotinic acetylcholine receptors. *BMC Cancer* 15**,** 152.

Chikova, A., and Grando, S.A. (2011). Naturally occurring variants of human Alpha9 nicotinic receptor differentially affect bronchial cell proliferation and transformation. *PLoS One* 6**,** e27978.

Dasgupta, P., Kinkade, R., Joshi, B., Decook, C., Haura, E., and Chellappan, S. (2006). Nicotine inhibits apoptosis induced by chemotherapeutic drugs by up-regulating XIAP and survivin. *Proc Natl Acad Sci U S A* 103**,** 6332-6337.

Fararjeh, A.S., Tu, S.H., Chen, L.C., Cheng, T.C., Liu, Y.R., Chang, H.L., Chang, H.W., Huang, C.C., Wang, H.R., Hwang-Verslues, W.W., Wu, C.H., and Ho, Y.S. (2019). Long-term exposure to extremely low-dose of nicotine and 4-(methylnitrosamino)-1-(3-pyridyl)-1-butanone (NNK) induce non-malignant breast epithelial cell transformation through activation of the a9-nicotinic acetylcholine receptor-mediated signaling pathway. *Environ Toxicol* 34**,** 73-82.

Guha, P., Bandyopadhyaya, G., Polumuri, S.K., Chumsri, S., Gade, P., Kalvakolanu, D.V., and Ahmed, H. (2014). Nicotine promotes apoptosis resistance of breast cancer cells and enrichment of side population cells with cancer stem cell-like properties via a signaling cascade involving galectin-3, alpha9 nicotinic acetylcholine receptor and STAT3. *Breast Cancer Res Treat* 145**,** 5-22.

Guo, J., Ibaragi, S., Zhu, T., Luo, L.Y., Hu, G.F., Huppi, P.S., and Chen, C.Y. (2008). Nicotine promotes mammary tumor migration via a signaling cascade involving protein kinase C and CDC42. *Cancer Res* 68**,** 8473-8481.

Huang, L.C., Lin, C.L., Qiu, J.Z., Lin, C.Y., Hsu, K.W., Tam, K.W., Lee, J.Y., Yang, J.M., and Lee, C.H. (2017). Nicotinic Acetylcholine Receptor Subtype Alpha-9 Mediates Triple-Negative Breast Cancers Based on a Spontaneous Pulmonary Metastasis Mouse Model. *Front Cell Neurosci* 11**,** 336.

Improgo, M.R., Schlichting, N.A., Cortes, R.Y., Zhao-Shea, R., Tapper, A.R., and Gardner, P.D. (2010). ASCL1 regulates the expression of the CHRNA5/A3/B4 lung cancer susceptibility locus. *Mol Cancer Res* 8**,** 194-203.

Kalantari-Dehaghi, M., Parnell, E.A., Armand, T., Bernard, H.U., and Grando, S.A. (2015). The nicotinic acetylcholine receptor-mediated reciprocal effects of the tobacco nitrosamine NNK and SLURP-1 on human mammary epithelial cells. *Int Immunopharmacol* 29**,** 99-104.

Kolodziej, M.A., Gott, H., Kopischke, B., Bender, M.K.F., Weigand, M.A., Di Fazio, P., Schwarm, F.P., and Uhle, F. (2021). Antiproliferative effect of GTS-21 in glioblastoma cells. *Oncol Lett* 22**,** 759.

Lam, D.C., Girard, L., Ramirez, R., Chau, W.S., Suen, W.S., Sheridan, S., Tin, V.P., Chung, L.P., Wong, M.P., Shay, J.W., Gazdar, A.F., Lam, W.K., and Minna, J.D. (2007). Expression of nicotinic acetylcholine receptor subunit genes in non-small-cell lung cancer reveals differences between smokers and nonsmokers. *Cancer Res* 67**,** 4638-4647.

Lee, C.H., Chang, Y.C., Chen, C.S., Tu, S.H., Wang, Y.J., Chen, L.C., Chang, Y.J., Wei, P.L., Chang, H.W., Chang, C.H., Huang, C.S., Wu, C.H., and Ho, Y.S. (2011). Crosstalk between nicotine and estrogen-induced estrogen receptor activation induces alpha9-nicotinic acetylcholine receptor expression in human breast cancer cells. *Breast Cancer Res Treat* 129**,** 331-345.

Lee, C.H., Huang, C.S., Chen, C.S., Tu, S.H., Wang, Y.J., Chang, Y.J., Tam, K.W., Wei, P.L., Cheng, T.C., Chu, J.S., Chen, L.C., Wu, C.H., and Ho, Y.S. (2010). Overexpression and activation of the alpha9-nicotinic receptor during tumorigenesis in human breast epithelial cells. *J Natl Cancer Inst* 102**,** 1322-1335.

Lin, C.Y., Lee, C.H., Chuang, Y.H., Lee, J.Y., Chiu, Y.Y., Wu Lee, Y.H., Jong, Y.J., Hwang, J.K., Huang, S.H., Chen, L.C., Wu, C.H., Tu, S.H., Ho, Y.S., and Yang, J.M. (2019). Membrane protein-regulated networks across human cancers. *Nat Commun* 10**,** 3131.

Mucchietto, V., Fasoli, F., Pucci, S., Moretti, M., Benfante, R., Maroli, A., Di Lascio, S., Bolchi, C., Pallavicini, M., Dowell, C., Mcintosh, M., Clementi, F., and Gotti, C. (2018). alpha9- and alpha7-containing receptors mediate the pro-proliferative effects of nicotine in the A549 adenocarcinoma cell line. *Br J Pharmacol* 175**,** 1957-1972.

Nguyen, H.D., Liao, Y.C., Ho, Y.S., Chen, L.C., Chang, H.W., Cheng, T.C., Liu, D., Lee, W.R., Shen, S.C., Wu, C.H., and Tu, S.H. (2019). The alpha9 Nicotinic Acetylcholine Receptor Mediates Nicotine-Induced PD-L1 Expression and Regulates Melanoma Cell Proliferation and Migration. *Cancers (Basel)* 11.

Pucci, S., Fasoli, F., Moretti, M., Benfante, R., Di Lascio, S., Viani, P., Daga, A., Gordon, T.J., Mcintosh, M., Zoli, M., Clementi, F., and Gotti, C. (2021a). Choline and nicotine increase glioblastoma cell proliferation by binding and activating alpha7- and alpha9- containing nicotinic receptors. *Pharmacol Res* 163**,** 105336.

Qian, J., Liu, Y.Q., Sun, Z.H., Zhangsun, D.T., and Luo, S.L. (2019). Identification of nicotinic acetylcholine receptor subunits in different lung cancer cell lines and the inhibitory effect of alpha-conotoxin TxID on lung cancer cell growth. *Eur J Pharmacol* 865**,** 172674.

Shih, Y.L., Liu, H.C., Chen, C.S., Hsu, C.H., Pan, M.H., Chang, H.W., Chang, C.H., Chen, F.C., Ho, C.T., Yang, Y.Y., and Ho, Y.S. (2010). Combination treatment with luteolin and quercetin enhances antiproliferative effects in nicotine-treated MDA-MB-231 cells by down-regulating nicotinic acetylcholine receptors. *J Agric Food Chem* 58**,** 235-241.

Spina, R., Voss, D.M., Asnaghi, L., Sloan, A., and Bar, E.E. (2016). Atracurium Besylate and other neuromuscular blocking agents promote astroglial differentiation and deplete glioblastoma stem cells. *Oncotarget* 7**,** 459-472.

Sun, Z., Zhangsun, M., Dong, S., Liu, Y., Qian, J., Zhangsun, D., and Luo, S. (2020a). Differential Expression of Nicotine Acetylcholine Receptors Associates with Human Breast Cancer and Mediates Antitumor Activity of alphaO-Conotoxin GeXIVA. *Mar Drugs* 18.

Sun, Z., Bao, J., Zhangsun, M., Dong, S., Zhangsun, D., and Luo, S. (2020b). alphaO-Conotoxin GeXIVA Inhibits the Growth of Breast Cancer Cells via Interaction with alpha9 Nicotine Acetylcholine Receptors. *Mar Drugs* 18.

Terpinskaya, T.I., Osipov, A.V., Balashevich, T.V., Yanchanka, T.L., Tamashionik, E.A., Tsetlin, V.I., and Utkin, Y.N. (2020). Blockers of Nicotinic Acetylcholine Receptors Delay Tumor Growth and Increase Antitumor Activity of Mouse Splenocytes. *Dokl Biochem Biophys* 491**,** 89-92.

Thompson, E.G., and Sontheimer, H. (2019). Acetylcholine Receptor Activation as a Modulator of Glioblastoma Invasion. *Cells* 8.

Tsurutani, J., Castillo, S.S., Brognard, J., Granville, C.A., Zhang, C., Gills, J.J., Sayyah, J., and Dennis, P.A. (2005). Tobacco components stimulate Akt-dependent proliferation and NFkappaB-dependent survival in lung cancer cells. *Carcinogenesis* 26**,** 1182-1195.

Tu, S.H., Ku, C.Y., Ho, C.T., Chen, C.S., Huang, C.S., Lee, C.H., Chen, L.C., Pan, M.H., Chang, H.W., Chang, C.H., Chang, Y.J., Wei, P.L., Wu, C.H., and Ho, Y.S. (2011). Tea polyphenol (-)-epigallocatechin-3-gallate inhibits nicotine- and estrogen-induced alpha9-nicotinic acetylcholine receptor upregulation in human breast cancer cells. *Mol Nutr Food Res* 55**,** 455-466.

Tu, S.H., Lin, Y.C., Huang, C.C., Yang, P.S., Chang, H.W., Chang, C.H., Wu, C.H., Chen, L.C., and Ho, Y.S. (2016). Protein phosphatase Mg2+/Mn2+ dependent 1F promotes smoking-induced breast cancer by inactivating phosphorylated-p53-induced signals. *Oncotarget* 7**,** 77516-77531.

West, K.A., Brognard, J., Clark, A.S., Linnoila, I.R., Yang, X., Swain, S.M., Harris, C., Belinsky, S., and Dennis, P.A. (2003). Rapid Akt activation by nicotine and a tobacco carcinogen modulates the phenotype of normal human airway epithelial cells. *J Clin Invest* 111**,** 81-90.
